# From source to sink: part 1—characterization and Lagrangian tracking of riverine microplastics in the Mediterranean Basin

**DOI:** 10.1007/s11356-024-34635-6

**Published:** 2024-08-17

**Authors:** Lisa Weiss, Claude Estournel, Patrick Marsaleix, Guillaume Mikolajczak, Mel Constant, Wolfgang Ludwig

**Affiliations:** 1 Univ. Toulouse, IRD, CNRS, CNES, UPS, Laboratoire d’Etudes en Géophysique et Océanographie Spatiales (LEGOS), Toulouse, 31400 France; 2 Univ. Perpignan Via Domitia, CNRS, Centre de Formation et de Recherche sur les Environnements Méditerranéens (CEFREM), Perpignan, 66000 France; 3 Univ. Lille, Institut Mines-Télécom, Univ. Artois, Junia, Laboratoire de Génie Civil et géo-Environnement (LGCgE), Lille, 59000 France

**Keywords:** Riverine microplastics, Mediterranean Basin, Lagrangian dispersion, Source to sink

## Abstract

**Abstract:**

The Mediterranean Sea is one of the most critically polluted areas due to its semi-enclosed structure and its highly anthropized shoreline. Rivers are significant vectors for pollutant transfers from the continental to the marine environment. In this context, a 3D Lagrangian simulation of the dispersion of riverine microplastics (MPs) was performed, which included the application of a recently developed model that reassessed the MP fluxes discharged by rivers. MP physical properties from river samples were further investigated to approximate vertical displacement in modeled ocean currents. The use of a high-resolution circulation model, integrating Stokes drift, turbulent diffusion, and MP sinking and rising velocities, enabled us to establish stock balances. Our simulation suggested that 65% of river inputs may be made of floating MPs drifting in the surface layer and 35% of dense MPs sinking to deeper layers. The Eastern Mediterranean tends to accumulate floating MPs, primarily originating from the Western Mediterranean Basin, where major river sources are concentrated. After 2 years of simulation, modeled stranding sequestered 90% of the MP inputs, indicating relatively short average residence times from a few days to months at most for particles at sea. Although spatial distribution patterns stabilized after this period and a steady state may have been approached, the surface concentrations we modeled generally remained below field observations. This suggested either an underestimation of sources (rivers and unaccounted sources), by a factor of 6 at most, or an overestimation of MP withdrawal through stranding, to be reduced from 90 to around 60% or less if unaccounted sinks were considered.

**Graphical abstract:**

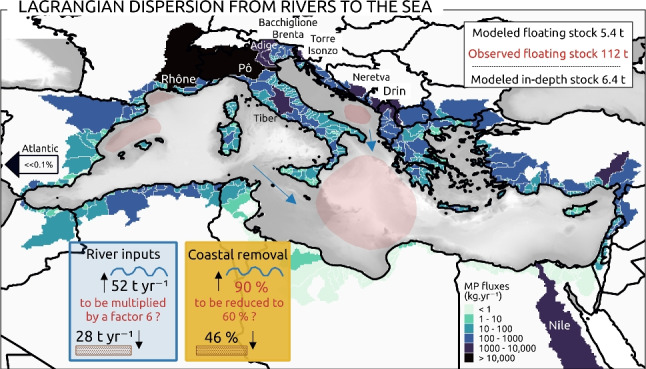

## Highlights

- The characterization of MP particles, based on observed shape, size, and density distributions, resulted in 65% floating and 35% sinking MPs.
- Riverine MP sources to the Mediterranean were estimated at around 80 $t \ year^{-1}$ or 682 billion MPs $year^{-1}$ (the ten main rivers accounted for 52%).
- Amount of MPs released by rivers was estimated 1 to 2 orders of magnitude higher in river basins from the north shore than from the south shore.
- Net MP exports occurred from the North-Western basin ($\sim$45% of its floating river sources) and Adriatic Sea (16%) to the Ionian Sea (increasing 2 times its river sources) and southern Levantin basin (increasing 1/4 of its river sources).
- Maximum average residence times at the sea surface ranged from 1 to 3 weeks in highly dissipative sub-basins to 11 weeks in the Ionian Sea.
- To match observed sea surface stocks ($\sim$310 billion floating MPs or $\sim$112 tons), unaccounted sources such as fragmentation should be introduced, strandings reduced around 60% and unconsidered sinks added.
- Almost half (46%) of the sinking MPs were stranded at the coast, and the other half (48%) remained on continental shelves between 50 and 500 m depth.

## Introduction

Environmental concerns about microplastics (MPs) in the oceans are still growing (Andrady 2017) and motivate numerous studies on their quantification and transfers to achieve global mass balances (Kaandorp et al. 2023; Harris et al. 2023; Sonke et al. 2022). This however requires a better understanding of the plastic sources, transport processes, and sinks in the marine environment. To this end, various ocean models have been used to simulate Lagrangian trajectories of particles in current fields. While on a global scale they succeed in reproducing approximately the accumulation of floating MPs observed in subtropical ocean gyres under the effect of Ekman transport (Bajon et al. 2023; Chenillat et al. 2021; Onink et al. 2019; van Sebille et al. 2015), accumulation scenarios are much more variable at smaller basin scales such as the Mediterranean where the distribution of MPs is more influenced by initial source configuration and transient processes such as river inputs, stranding, current velocities, waves, or wind directions (Tsiaras et al. 2022). Previous Lagrangian models in the Mediterranean Sea (Baudena et al. 2023; Fabri-Ruiz et al. 2023; Guerrini et al. 2022; Hatzonikolakis et al. 2022; Soto-Navarro et al. 2020; Macias et al. 2019, 2022; Liubartseva et al. 2018; Zambianchi et al. 2017) all initialized their dispersion scenarios with riverine inputs derived from mismanaged plastic waste (MPW) models (Jambeck et al. 2015; Lebreton et al. 2017) leading to the simulation of floating stocks much higher than observed concentrations. Kaandorp et al. (2020), using an inverse modeling method parameterized with field measurements of plastic concentrations at sea, are the first authors to question this, leading them to reassess the source and sink budgets to much lower values.

Moreover, most studies consider 2D approaches where neutral Lagrangian particles drift on the sea surface without considering their vertical velocity. The dynamic behavior of plastic particles related to their highly variable physical and chemical properties as well as the turbulent surface layer conditions is thus ignored. Liubartseva et al. (2018) and Kaandorp et al. (2020), for example, integrated particle sedimentation into a 2D Mediterranean-scale modeling study using a probability criterion based on surface residence times. So far, only Soto-Navarro et al. (2020), Hatzonikolakis et al. (2022), and Baudena et al. (2023) considered a 3D dispersion of particles in the Mediterranean. The first authors distinguished three classes of particles, i.e., neutral following the water masses, floating at the surface, and dense with a constant sinking velocity equal to $10^{-3} \ m \ s^{-1}$. The second authors integrated a progressive loss of buoyancy through biofouling as a function of residence time, bacterial abundance, and debris size. In order to fill these gaps, tools such as *TrackMPD* (Jalón-Rojas et al. 2019) were recently developed to integrate different physical processes associated with MPs into hydrodynamic models, in order to test the impact of winds, sinking rates, and stranding and biofouling on MP transport. Nevertheless, better model parametrization requires more precise knowledge of the characteristics of the MPs in rivers, one of the main vectors of contaminants from land to the sea.

The first objective of this study is to present a new empirical method for characterizing virtual particles in Lagrangian dispersion models to generate more diverse and variable trajectories, based on in situ observations in rivers and experimental studies. For this purpose, a compilation of bibliographic information on the main physical properties of MPs collected in rivers was performed to determine representative statistical distributions of sizes, shapes, and densities of MPs released at sea. These distributions were then used to calculate sinking and rising velocities from empirical equations (Khatmullina and Isachenko 2017; Zhiyao et al. 2008).

The second objective is to apply a recently developed global flux model of riverine MPs (Weiss et al. 2021) to the Mediterranean Basin in order to re-evaluate and re-distribute the MP fluxes discharged by Mediterranean rivers. This allowed the Lagrangian simulations to be performed based on a completely novel spatial and quantitative initialization of source scenarios. Considering the various processes inherent to plastics at sea, this study has therefore prioritized the investigation of the river sources and the principal characteristics of river MPs. Simulations of ocean circulation and Lagrangian trajectories were performed with the 3D hydrodynamic model SYMPHONIE (Marsaleix et al. 2006, 2008) over the entire Mediterranean Basin. Its Lagrangian tracking module has been modified and adapted to MP transport in order to integrate the MP characterization described above. MP trajectories were calculated on a kilometer telescopic grid resolution (2 to 4.5 km), with Eulerian currents forced by the wave-related Stokes drift and Lagrangian particles associated with a wide range of sinking and rising velocities.

The third objective is to analyze the MP distribution in the surface layer and at depth, as well as the MP concentrations in the different Mediterranean sub-basins. This analysis was used to establish a new regional source-to-sink budget from rivers to stranding, in the light of previous and most recent observations.

**Table 1:** Density ranges of the most frequently observed polymers considered in this study. The number of particles per shape and type of polymer considered in this study are derived from the field studies of van der Wal et al. (2015) and Constant et al. (2020)

Polymers and density ($g\ cm^{-3}$)	nb of fragments	Beads	Fibers	Foams identified
Polyethylene (PE) 0.9–0.96	772	123	4	2
Polypropylene (PP) 0.9–0.91	144	49	23	0
Polystyrene (PS) 1.04–1.1	9	7	0	0
Expanded polystyrene (EPS) 0.01–0.05	0	0	0	79
Polyethylene terephthalate (PET) 1.24–1.45	2	0	28	0
Polyvinyl chloride (PVC) 1.16–1.58	2	0	0	0
Polyurethane (PU) 1–1.26	0	0	0	2
Polyamide (PA) 1.02–1.16	5	0	8	0
Polyvinyl acetate (PVA) 1.09–1.3	3	1	10	2
Other	16	7	12	9

## Method

### Observed MP characteristics

#### Size distribution

**Fig. 1:**
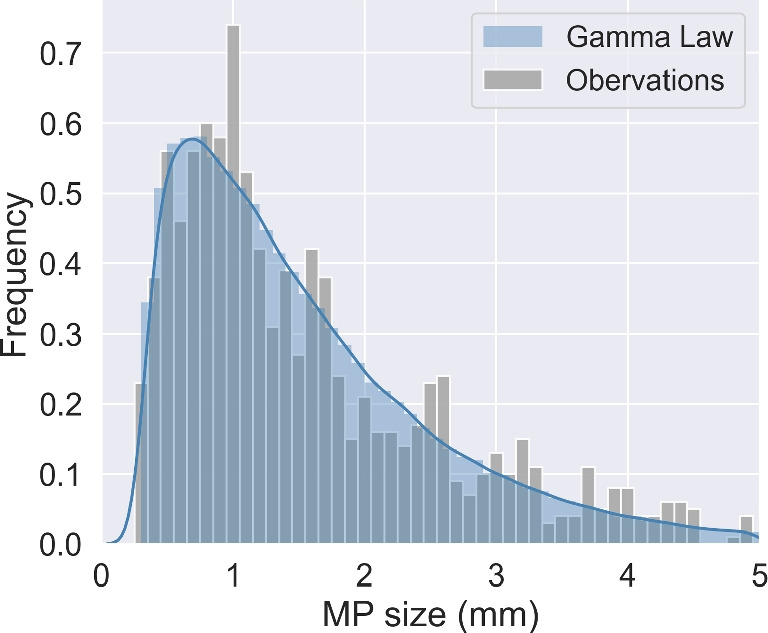
MP size histograms observed in Japan rivers by Kataoka et al. (2019) (in gray) and the associated probability density function following a gamma law (in blue)

We considered in the study MPs ranging in size from 0.3 *mm*, the most usual net mesh size for MP sampling (Weiss et al. 2021), to 5 *mm*, the standard upper limit (GESAMP 2019). We defined a particle size distribution that best approximated the most detailed size observations in rivers we found in the literature, from the study of Kataoka et al. (2019) who sampled MPs in Japan rivers with 300 $\mu m$ plankton nets (gray histogram in Fig. 1 based on 804 individual MPs measured). Then, a random draw of MP sizes was carried out following the gamma probability density function fitted to the observed distribution. This generated a histogram of MP sizes over the interval 0.3–5 *mm* to configure the daily release of Lagrangian particles in the simulation.

#### Polymer types and densities

The MP density depends mainly on the type of polymer that constitutes the particle, ranging generally from 0.9 to 1.2 $g \ cm^{-3}$ in plankton net samples (Chubarenko et al. 2016, and Table 1). As a comparison, the density of freshwater is equal to 1 $g \ cm^{-3}$ and that of Mediterranean seawater averaged to 1.028 $g \ cm^{-3}$. Despite the great diversity of existing plastics, 6 polymers account for more than 90% of the world production: PE, PP, PVC, PET, PU, and PS/EPS (Geyer et al. 2017; see Table 1 for acronyms). To produce a representative distribution of polymer types carried by rivers, we processed MP chemical composition analyses provided by two field studies in five European rivers: the Rhine (6 samplings), the Po (3 samplings), the Danube (3 samplings) by van der Wal et al. (2015), the Rhone (28 samplings), and the Têt (35 samplings) rivers by Constant et al. (2020). All data were sampled with 300 µm plankton nets. Both studies provided the number of MPs per polymer type and shape for all the samplings. For each shape, we calculated the percentage of each polymer type represented in each sampling and then all samplings combined (Fig. 3d). The overall averages encompassing all rivers and shapes were then obtained (Fig. 3b).

#### Shape distributions

Shape characteristics also affect the buoyancy of MPs (Isachenko et al. 2016). Here, we considered four MP shapes, e.g., fragments, fibers, beads, and foams. The relative proportion of each shape was determined by averaging data from several studies that provided MP shape distributions in river plankton net samples (Table 2, 0.3 to 5 *mm*). Accordingly, foams and beads were characterized in empirical equations by their diameter, fragments by their characteristic size equal to the cube root of the longest, intermediate, and shortest axis, and fibers by their diameter and length.

**Table 2:** Shape distributions (%) in literature studies

References	Rivers	Fragments	Beads	Fibers	Foams
van der Wal et al. (2015)	Danube (3 sampling)	9.6	0.6	76.2	13.7
van der Wal et al. (2015)	Po (3 sampling)	56.3	10.4	17.7	15.7
van der Wal et al. (2015)	Rhine (6 sampling)	51.8	27.5	10	10.7
Constant et al. (2020)	Rhone	1.7	0.1	97.9	0.3
Constant et al. (2020)	Têt	5.6	0.7	92.5	1.2
Simon-Sánchez et al. (2019)	Ebro	52.9	0.5	46.1	0.5
Tan et al. (2019)	Feilaixia s1–s6	73.2	0	23.1	3.8
Baldwin et al (2016)	Great lakes tributaries	20	2	71	7
Faure et al. (2015)	Swiss rivers	75	6	8	11
Mani et al. (2015)	Rhine	37.5	58.4	2.5	1.6
Campbell et al. (2017)	Wascana creek	10.2	0.3	89.5	0
Mean		35	10	50	5

#### MP vertical velocities

The sinking velocity ($w_s$) of a particle in a fluid can be modeled by balancing the buoyancy force with the hydrodynamic drag force (Stokes 1851) following Eq. 1, and depends mainly on the three physical properties described above: size, density, and shape (Khatmullina and Isachenko 2017; Chubarenko et al. 2016; Kooi et al. 2016).

1 $$\begin{aligned} \frac{1}{2} \ \rho \ C_D \ S \ w_s^{2} \ = \ (\rho _p - \rho _w) \ V g \end{aligned}$$

$C_D$ is the drag coefficient, *S* and *V* are the projected area and volume of the particle, $\rho _p$ is the particle density, $\rho _w$ is the fluid density, and *g* is the gravity acceleration ($mm \ s^{-2}$). We used existing experimental studies that expressed $w_s$ based on Eq. 1 and the empirical determination method of $C_D$. Since the shape influences the MP vertical motion, we considered two different semi-empirical equations to calculate $w_s$ (as done by Constant et al. 2023).

On the one hand, beads and foams were assimilated into spheres and fragments into flat particles of variable proportions using the formula published by Zhiyao et al. (2008) (Eq. 2). The applicability of their formula based on sedimentary studies to various MPs was verified by several experimental studies, which revealed a good fit for spherical and cylindrical MPs, but not for synthetic fibers (Chubarenko et al. 2016; Isachenko et al. 2016; Khatmullina and Isachenko 2017; Waldschläger and Schüttrumpf 2019).

2 $$\begin{aligned} w_s \ = \frac{g \ (\rho _p - \rho _w)}{d \ \nu \ \rho _w} \ d^3 \ [38.1 + 0.93 \ d_*^{\frac{12}{7}}]^{-\frac{7}{8}} \ (mm \ s^{-1}) \end{aligned}$$

*d* corresponds to the diameter for beads and to the characteristic size equal to the cube root of the longest, intermediate, and shortest axis for fragments (all in [0.3, 5] *mm*), $\rho _w$ was set at 1028 $kg \ m^{-3}$ and the kinematic viscosity of the fluid $\nu = \frac{\eta }{\rho _w} \approx \frac{1.07 \ 10^{-3} \ kg \ m^{-1} s^{-1}}{1028 \ kg \ m^{-3}} \approx 1.041 \ mm^{2} \ s^{-1}$ for Mediterranean Sea water.

On the other hand, synthetic fibers were related to cylinders of variable proportions based on the formula of Khatmullina and Isachenko (2017) (Eq. 3) with *D* the diameter in [0.03, 0.5] *mm*, *L* the characteristic length in [0.3, 5] *mm* and the empirical coefficients $c_1 = 55.238 \ mm^{-1}$ and $c_2 = 12.69$.

3 $$\begin{aligned} w_s = \frac{\pi }{2 \nu } \frac{g \ (\rho _p - \rho _w)}{\rho _w} \frac{D L}{c_1 L + c_2} \ (mm \ s^{-1}) \end{aligned}$$

We used the same two semi-empirical equations, to calculate both rising and sinking velocities, which differed only in their mathematical sign. Thus, MPs with polymer density lower than the surrounding water were characterized by positive $w_s$, i.e., rising velocities oriented towards the surface. MPs with polymer density higher than the surrounding water were characterized by negative $w_s$, i.e., sinking velocities oriented towards the seafloor.

### Source and sink of MPs in the sea

#### River MP fluxes

The MP mass fluxes released annually by rivers into the sea were calculated using the empirical regression model *plankton net* (Eq. 4) published by Weiss et al. (2021), driven by population density (*sPoP*) and water runoff (*sQ*) in the corresponding river basins:

4 $$\begin{aligned} sF_m = 5.09.10^{-6} \ sPop^{0.339} \ sQ^{1.32} \end{aligned}$$

This model corrected methodological errors made in previous estimates and reassessed river MP fluxes globally. It was applied to each of the 549 Mediterranean river basins delineated by Sadaoui et al. (2018) at a high spatial resolution of 0.08$^\circ$. The resulting specific mass fluxes ($sF_m$ in $kg \ km^{-2} year^{-1}$) were then transformed to absolute mass fluxes ($F_m$ in $kg \ year^{-1}$) on the basis of the river basin areas. The averaged population densities inside the basins (*sPop* in $inhabitants \ km^{-2}$) were extracted from the CIESIN (2018) gridded data set and the runoff (*sQ* in $mm \ year^{-1}$), were interpolated from Ludwig et al. (2009).

For the purpose of our Lagrangian tracking simulation, mass fluxes had to be converted in number fluxes ($F_p$ in MPs $year^{-1}$), considering separately synthetic fibers from other MP shapes due to their significantly different mean masses (Eq. 5). To this end, we used the conversion factors from Weiss et al. (2021) equal to 0.745 $\mu g$ for fibers and 0.233 *mg* for non-fiber MPs. $P_f$ is the proportion of fibers to be considered in the study, based on shape distribution in samples (50% in this study, Table 2).

5 $$\begin{aligned} F_p = \frac{F_m}{P_f \ 0.745.10^{-9} + (1 - P_f) \ 0.233.10^{-6}} \end{aligned}$$

The release position for each particle was randomly drawn within a 0.04$^\circ$ square area delineated off each of the 549 Mediterranean river mouths with a corresponding relative MP flux ($F_p$). Notice that rivers were the only sources in our study. The contributions of the Atlantic and the Black Sea were not included, nor were the direct inputs from coastal cities and leakage from marine traffic. To save computational time, we considered that 1 Lagrangian particle in the simulation represented 10,000 MPs estimated by the river input model. The data processing presented in the following is thus expressed in the number of MPs, taking into account this 1:10,000 conversion.

We estimated the total MP river input into the Mediterranean Sea to be about 80 $t \ year^{-1}$ (Eq. 4). According to the conversion factors (Eq. 5), this mass flux represented about 682 billion MPs $year^{-1}$, i.e., 1.8 billion MPs $day^{-1}$. Therefore, a total of 886 billion floating MPs and 478 billion dense MPs were considered here with continuous daily release over 2 years (2013 and 2014) at river mouths. The simulation lasts until 2016.

#### Sinks configuration

Particle sequestration in the sediment was not parameterized in our configuration. MPs cannot settle on the bottom and were kept in the first water grid cell above the seafloor, where currents were usually very slow in such micro-tidal regions. We did not consider degradation processes, biofouling, or ingestion by organisms. When particles reached the coast, i.e., the edge of the last water grid cells where currents were very weak, they could remain motionless for very long periods. Although regional models such as ours on the Mediterranean Sea have a kilometer resolution that does not permit to finely resolve coastal processes, we did not add stochastic criteria, as was done by Liubartseva et al. (2018) and Kaandorp et al. (2020), because no observations were available when we conducted our study to parameterize the stranding and retention times of MPs. Consequently, they were considered definitively stranded when they moved less than 100 *m* in 1 month of simulation. The assumption of definitive stranding was made because the negligible tides in the Mediterranean may limit the potential daily remobilization of stranded particles, but also because stranding has been considered in other modeling studies as the main MP sink in the Mediterranean Sea (Liubartseva et al. 2018; Macias et al. 2019; van Sebille et al. 2020; Tsiaras et al. 2022; Baudena et al. 2022). However, it is acknowledged that wind and wave actions, which are intensified during storms, can be significant processes for the remobilization of stranded plastics, leading to potential important turnover rates on Mediterranean beaches (Bowman et al. 1998; Baudena et al. 2022). Some authors assume that remobilized plastics tend to remain mostly in the coastal zone and strand again a few km further away (Zhang 2017; Isobe et al. 2014), suggesting that virtual MP resuspension in coastal grids of a few kilometers of resolution would not alter significantly the final simulated distribution of strandings. In our model, stranding can occur in any coast following the circulation; the diversity of coastlines is not taken into account.

### Hydrodynamic model

#### The model

The simulation was performed with the three-dimensional ocean circulation model SYMPHONIE (Marsaleix et al. 2006, 2008). This model solves the primitive equations for mass and momentum conservation and is based on Boussinesq approximations and hydrostatic equilibrium. SYMPHONIE has already been used at different scales in the Mediterranean Sea from the coastal scale (Estournel 2003; Michaud et al. 2012; Bouffard et al. 2008), to the mesoscale (Estournel et al. 2016) and sub-mesoscale (Damien et al. 2017) and for various applications such as deep convection, dense water formation, upwelling phenomena, biogeochemical cycling or sediment transport (Mikolajczak et al. 2020; Many et al. 2021; Ulses et al. 2021; Estournel et al. 2023). The physical simulation used here has recently been detailed and validated for the whole Mediterranean Basin by Estournel et al. (2021).

#### The grid

The grid is a telescopic Arakawa-C-grid with horizontal curvilinear coordinates (Estournel et al. 2021). The resolution is about 1.3 *km* at the Gibraltar strait and 2 *km* in the Gulf of Lion (Northwestern Mediterranean) and increases to 4.5 *km* in the extreme east of the Levantine basin (Southeastern Mediterranean). The grid has 60 VQS (vanishing quasi-sigma) vertical levels (Estournel et al. 2021). The bathymetry was built from the GEBCO database.

The use of a high-resolution grid allowed the representation of large-scale, mesoscale, and partially sub-mesoscale structures, in particular filaments and fronts that have been shown to be key areas for floating MP trapping. Few other studies were based on such high grid resolution (3 *km* for Soto-Navarro et al. 2020; Baudena et al. 2023; 6–9 *km* for Mansui et al. 2015, 2020; Liubartseva et al. 2018; Kaandorp et al. 2020; Fabri-Ruiz et al. 2023).

#### Forcing

The interface between the ocean and the atmosphere is a free surface boundary where atmospheric forcing was simulated with bulk formulae. The atmospheric variables, wind components, pressure, temperature, humidity, and solar/thermal radiations came from the analyses of the European Center for Medium-Range Weather Forecasts (ECMWF) with a spatial resolution of 0.125$^\circ$ and an hourly temporal resolution. The initial and open boundary conditions in the Atlantic Ocean were provided by the operational oceanography center Mercator Ocean International as described by Estournel et al. (2021). The lateral open boundary conditions were described by Marsaleix et al. (2006). Open boundary conditions at river mouths were described by Nguyen-Duy et al. (2021). The freshwater inflows were daily mean values for ten French rivers, including the Rhone River, and Ebro, Arno, and Po. For the other rivers, discharges were mean annual values or seasonal climatology.

#### Current-wave interaction

Fabri-Ruiz et al. (2023), Baudena et al. (2022), Pedrotti et al. (2022), and Kaandorp et al. (2020) integrated the Stokes drift by simple addition of the separately predicted Eulerian and Stokes currents, as opposed to online forcing used by Liubartseva et al. (2018). Online forcing is more rigorous since it creates the anti-Stokes force that retroactively modifies the Eulerian currents at each time step. This method generally reduces the currents obtained and therefore conditions the drift of plastics at the surface. We have therefore chosen this method. We used the Stokes drift component calculated by the WAVEWATCH III wave generation and propagation model (Tolman 2009) to force the low-frequency Eulerian currents from SYMPHONIE related to thermohaline gradients, wind, and tidal forcing. This simple one-way forcing inspired by McWilliams and Restrepo (1999) implies that the hydrodynamic is assumed to be more influenced by the Stokes drift than the wave characteristics are by the general circulation. This forcing has already been validated at coastal scales with SYMPHONIE (Michaud et al. 2012; Rétif 2015; Mikolajczak et al. 2020).

The total current (*u*, *v*) is the sum of the Eulerian velocities ($\hat{u}, \hat{v}$) and the Stokes velocities ($U_s, V_s$):

6 $$\begin{aligned} \left\{ \begin{matrix} u=\hat{u}+U_s \\ \\ v=\hat{v}+V_s \\ \end{matrix}\right. \end{aligned}$$

Thus, the general equations of the SYMPHONIE model are rewritten such as follows:

7 $$\begin{aligned} &  \frac{d \hat{u}}{d t} -f(\hat{v}+V_s) =-\frac{1}{\rho _0}\frac{\partial p}{\partial x} +\frac{\partial }{\partial z}(K_m\frac{\partial \hat{u}}{\partial z}) +\Delta ^2\hat{u}\end{aligned}$$

8 $$\begin{aligned} &  \frac{d \hat{v}}{d t} +f(\hat{u}+U_s) =-\frac{1}{\rho _0}\frac{\partial p}{\partial y} +\frac{\partial }{\partial z}(K_m\frac{\partial \hat{v}}{\partial z})+\Delta ^2\hat{v} \end{aligned}$$

9 $$\begin{aligned} &  \frac{d T}{d t} = \frac{\partial }{\partial z} (K_h \frac{\partial T}{\partial z}) + \Delta T + \frac{\partial }{\partial z} I\end{aligned}$$

10 $$\begin{aligned} &  \frac{d S}{d t} = \frac{\partial }{\partial z} (K_h \frac{\partial S}{\partial z}) + \Delta S\end{aligned}$$

11 $$\begin{aligned} &  \frac{\partial \eta }{\partial t}=-\frac{\partial }{\partial x} (\int _{-h}^{\eta }udz) - \frac{\partial }{\partial y} (\int _{-h}^{\eta }vdz) \end{aligned}$$

The total vertical current is deduced from the continuity equation. *f* is the Coriolis parameter, $\rho _0$ is the density of the reference seawater, *p* is the pressure, *T* the temperature, *S* the salinity, $\Delta$ and $\Delta ^2$ are horizontal Laplacian and Bilaplacian operators, $K_m$ and $K_h$ are the vertical turbulent diffusivity coefficients calculated by the turbulent closure scheme (k-epsilon), and *I* is a radiative forcing term. The surface elevation is given by the transport divergence in Eq. 11. The derivation operator of Eqs. 7 to 10 is written:

12 $$\begin{aligned} \frac{d}{dt}=\frac{\partial }{\partial t}+u\frac{\partial }{\partial x}+v\frac{\partial }{\partial y}+w\frac{\partial }{\partial z} \end{aligned}$$

**Fig. 2:**
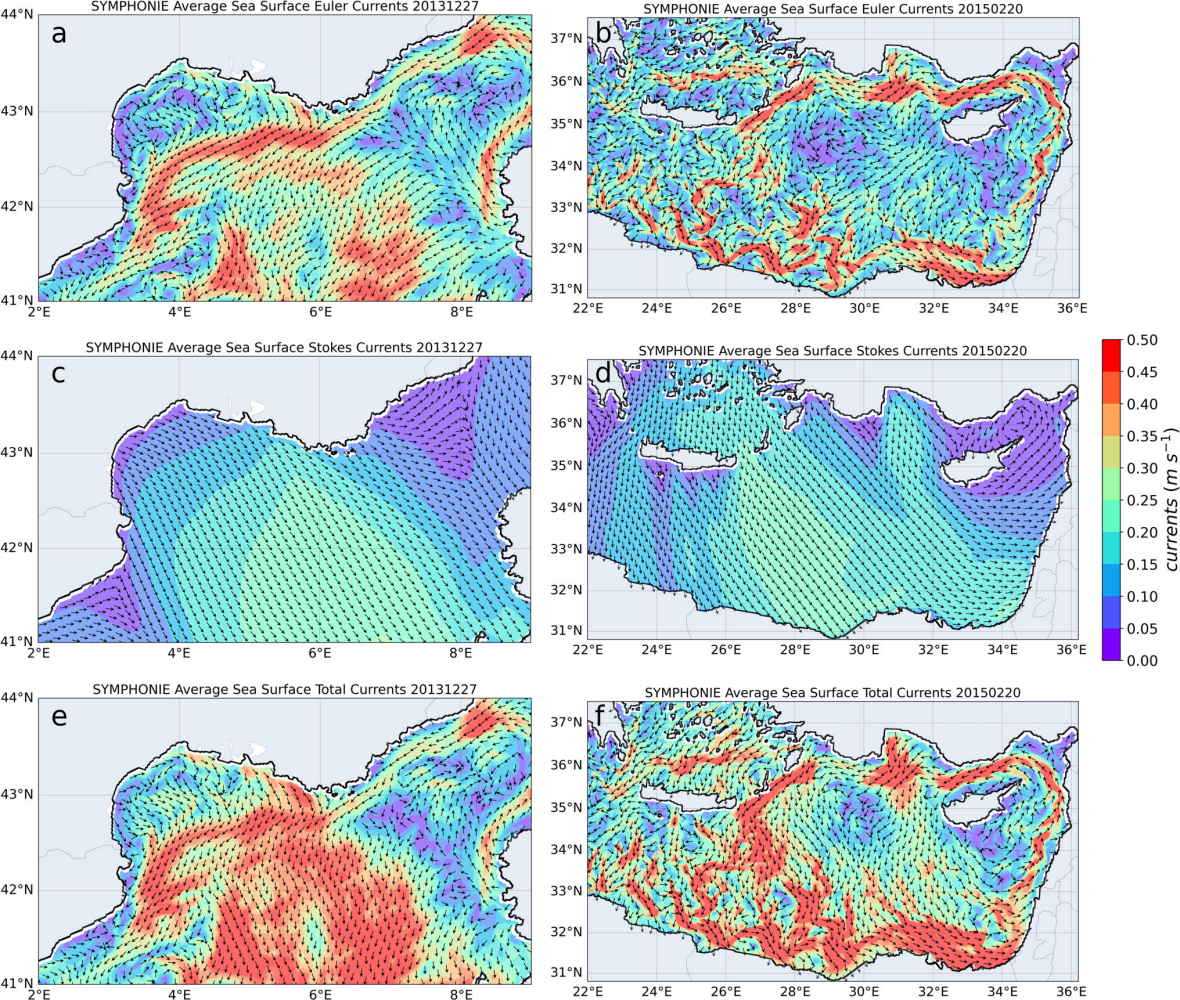
**a** and **b** Eulerian surface currents, **c** and **d** Stokes drift, and **e** and **f** total currents during a northwest-north wind event on December 27, 2013, in the Gulf of Lion (left) and February 20, 2015, in the Levantine Basin (right)

Although Stokes velocities are generally weaker than Eulerian currents, the effect of Stokes drift on surface currents during specific wind events can be significant, especially in winter when winds and river MP inputs are strongest. To illustrate this effect, Fig. 2 presents examples of wind events in the Levantine basin southeast of Crete and in the central western Mediterranean that can be considered representative. In the Gulf of Lion, north and northwestern winds correspond to Tramontane or Mistral events (Fig. 2 a, c, e). These winds generated high southward Stokes drift velocities, which also increased the southward current velocities. The export of floating particles was potentially favored in this same southerly direction. In the Levantine Basin (Fig. 2b, d, f), north and northwestern winds correspond to the Etesians. Through Stokes drift, they increased the southward currents resulting in an increase in current velocity along the southern coast and contributed to an intensification of the cyclonic circulation across the sub-basin.

### Lagrangian tracking

#### Lagrangian integration

Lagrangian integration was done through online trajectory calculations with the Lagrangian tracking module included in the hydrodynamic model SYMPHONIE. The integration time step of the particle trajectories is 780 s, and the calculation time step of the currents is 208 s. The online calculation mode allowed us to compute MP displacement with a higher frequency than the daily averaged storage usually done in the offline mode, considering for example inertial oscillations and internal waves that play a role in MP dispersion (van Sebille et al. 2020).

The time derivative of the 3D position vector $X(t) = (x, y, z)(t)$ is equal to the total current field calculated by the hydrodynamic model $U(t) = (u, v, w)(t)$, that describes the motion of the particles (Eq. 13). For the purposes of our study, the Lagrangian module has been modified to add a turbulent term $w_t$ and a buoyant term $w_s$ to the vertical current component *w* such as follows:

13 $$\begin{aligned} \frac{dX(t)}{dt} = U(t) {\left\{ \begin{array}{ll} \frac{dx(t)}{dt} = u(t) \\ \\ \frac{dy(t)}{dt} = v(t) \\ \\ \frac{dz(t)}{dt} = w'(t) = w(t) + w_t(t) + w_s(t) \end{array}\right. } \end{aligned}$$

$w_t$ represents the MP turbulent vertical diffusion, calculated at each integration time step of the particle trajectory (following Eq. 15). $w_s$ represents specific rising or sinking velocity, which were constants calculated in pre-processing (thanks to Eqs. 2, 3) and stored in a file read at the start of the simulation with the time and geographical location for each particle release.

#### Advective displacement

To solve the differential equations for the current velocities $U = (u, v, w)$ at the particle location, we used the two-stage second-order Runge–Kutta method (Eq. 14). RK2 is a good compromise between accuracy and total computation cost (i.e., when the cost of interpolating the velocity field at the particle location is also considered), especially when the time step used is less than the time required to cross a grid cell (our case). RK4 is only of interest if the current interpolation can restore the spatiotemporal non-linearities of the current field, which requires a very costly high-order current interpolation. The new position ($x_{n+1}, y_{n+1}, z_{n+1}$) of the particle is calculated such as follows:

14 $$\begin{aligned} \text {Step 1} \quad {\left\{ \begin{array}{ll} x^*_{n+1} = x_n + u(x_n,y_n,z_n) \Delta t \\ y^*_{n+1} = y_n + v(x_n,y_n,z_n) \Delta t \\ z^*_{n+1} = z_n + w'(x_n,y_n,z_n) \Delta t \nonumber \end{array}\right. } \\ \text {Step 2} \quad {\left\{ \begin{array}{ll} x_{n+1} = x_n + \frac{1}{2} (u(x_n,y_n,z_n) + u(x^*_{n+1},y^*_{n+1},z^*_{n+1})) \Delta t \\ y_{n+1} = y_n + \frac{1}{2} (v(x_n,y_n,z_n) + v(x^*_{n+1},y^*_{n+1},z^*_{n+1})) \Delta t \\ z_{n+1} = z_n + \frac{1}{2} (w'(x_n,y_n,z_n) + w'(x^*_{n+1},y^*_{n+1},z^*_{n+1})) \Delta t \end{array}\right. } \end{aligned}$$

where $w'$ contains the vertical current *w*, the specific vertical velocity traducing the buoyancy of the particle $w_s$ and the turbulent vertical diffusion $w_t$ (described hereafter).

#### Turbulent vertical diffusion

Turbulence in the surface layer, whether induced by wind, waves, or current shear, affects the vertical movement and the dispersion of floating MPs (Kukulka et al. 2012; Poulain et al. 2019). The surface mixed layer is about 10 to 100 *m* thick and can even reach a depth down to several hundred meters during convective periods in the Mediterranean. Understanding this MP vertical movement induced by several complex processes in the ocean surface layer remains difficult. In our study, we used a random-walk model (Eq. 15) to simulate the turbulent vertical diffusion as it was done for larval dispersal simulations by Guizien et al. (2012) (mechanistic approaches also exist (Brunner et al. 2015; Kukulka and Brunner 2015) based on wind stress analysis and subsurface observations). A turbulent vertical velocity $w_t$ was expressed as a function of the turbulent kinetic energy (*tke*) resolved by the hydrodynamic model and a random number $R_n$ between 0 and 1:

15 $$\begin{aligned} w_t = \sqrt{2 \frac{tke}{3}} \ 2\sqrt{3} (R_n - \frac{1}{2}) \end{aligned}$$

The normalization by $2\sqrt{3}$ in Eq. 15 allowed to obtain the average random kinetic energy equal to $\frac{tke}{3}$ over a large number of random draws.

The model prioritizes the turbulent vertical diffusion over the horizontal one due to a dominant vertical turbulence in the wind-influenced surface layer and stronger vertical current gradients. Horizontal disturbances would have a negligible impact on particle dispersion.

## Results

### MP physical properties

#### Shape, size, and polymers

**Fig. 3:**
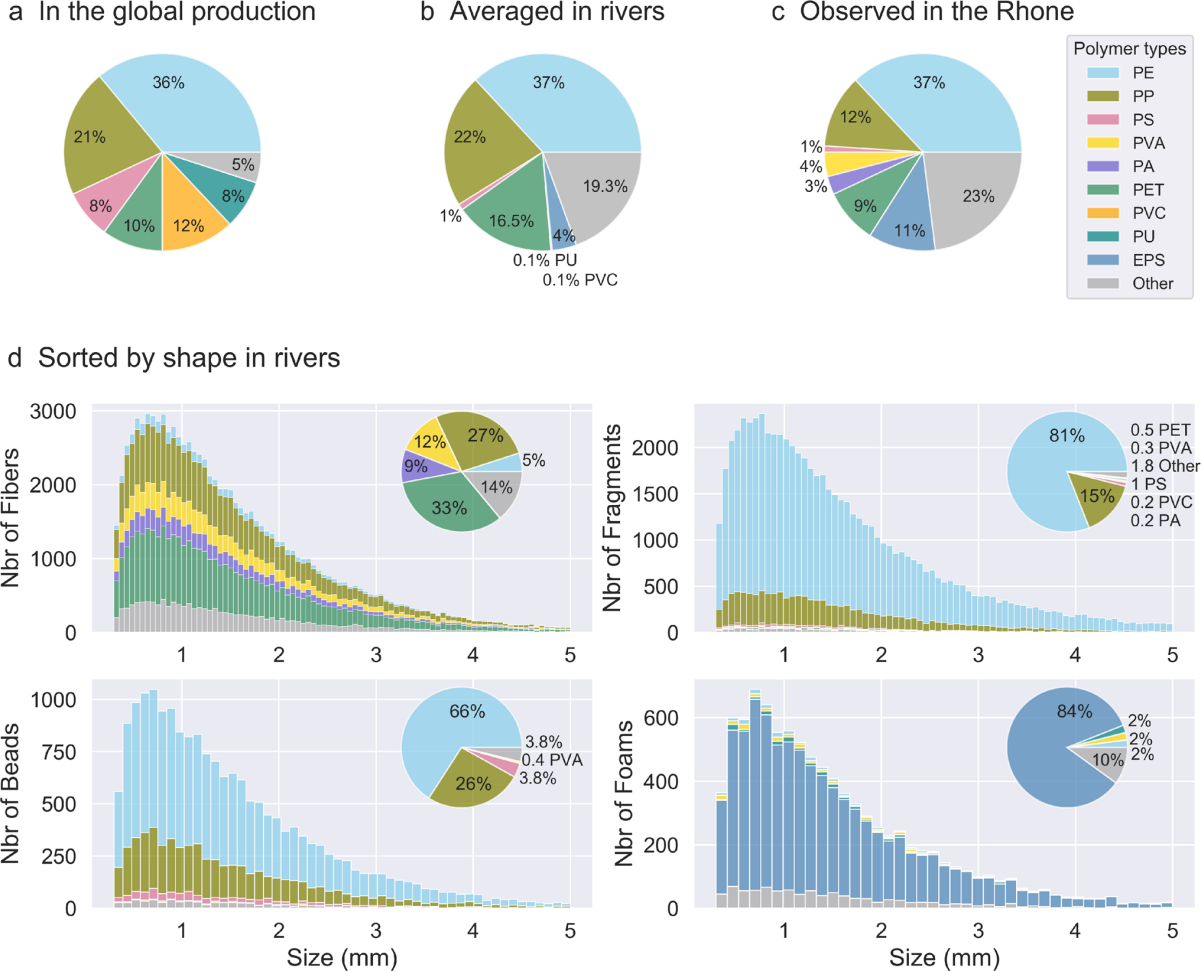
Relative distribution of the most common polymer types **a** in the global plastics mass production (based on Geyer et al. 2017, for Europe, USA, China, and India), **b** in European rivers (based on van der Wal et al. 2015; Constant et al. 2020), **c** in the Rhone River (Constant et al. 2020), and **d** in rivers sorted by shapes with size distribution

The average MP shape distribution obtained for rivers is 50% of synthetic fibers, 35% of fragments, 10% of beads, and 5% of foams. For density characterization, we observed that the most produced polymers (Fig. 3a) were also the most abundant MPs in rivers (Fig. 3b). The composition of the samples from the Rhone River (Fig. 3c), the largest individual freshwater contribution to the Mediterranean, was also similar to the average composition in European rivers. Figure 3 shows that PE and PP were the most predominant polymers, with a cumulative proportion generally higher than 50%, due to their low density between 0.9 and 0.96 $g \ cm^{-3}$ (Table 1), which makes them easier to float and trap in surface trawls. High-density polymers like PS, PET, PVC, PU, PVA, or PA were also present, with a relative predominance of PET. The observed MP size distribution following a gamma law showed a prevalence of small MPs $<1mm$ due to degradation and fragmentation processes leading to an exponential increase in the number of particles in the smaller sizes. However, a decrease in the number of MPs was observed for the size classes close to 300 $\mu m$ because the smaller the debris is, the greater is the probability that they pass through plankton net meshes or escape from visual counting (Fig. 3d).

#### Vertical velocities

**Fig. 4:**
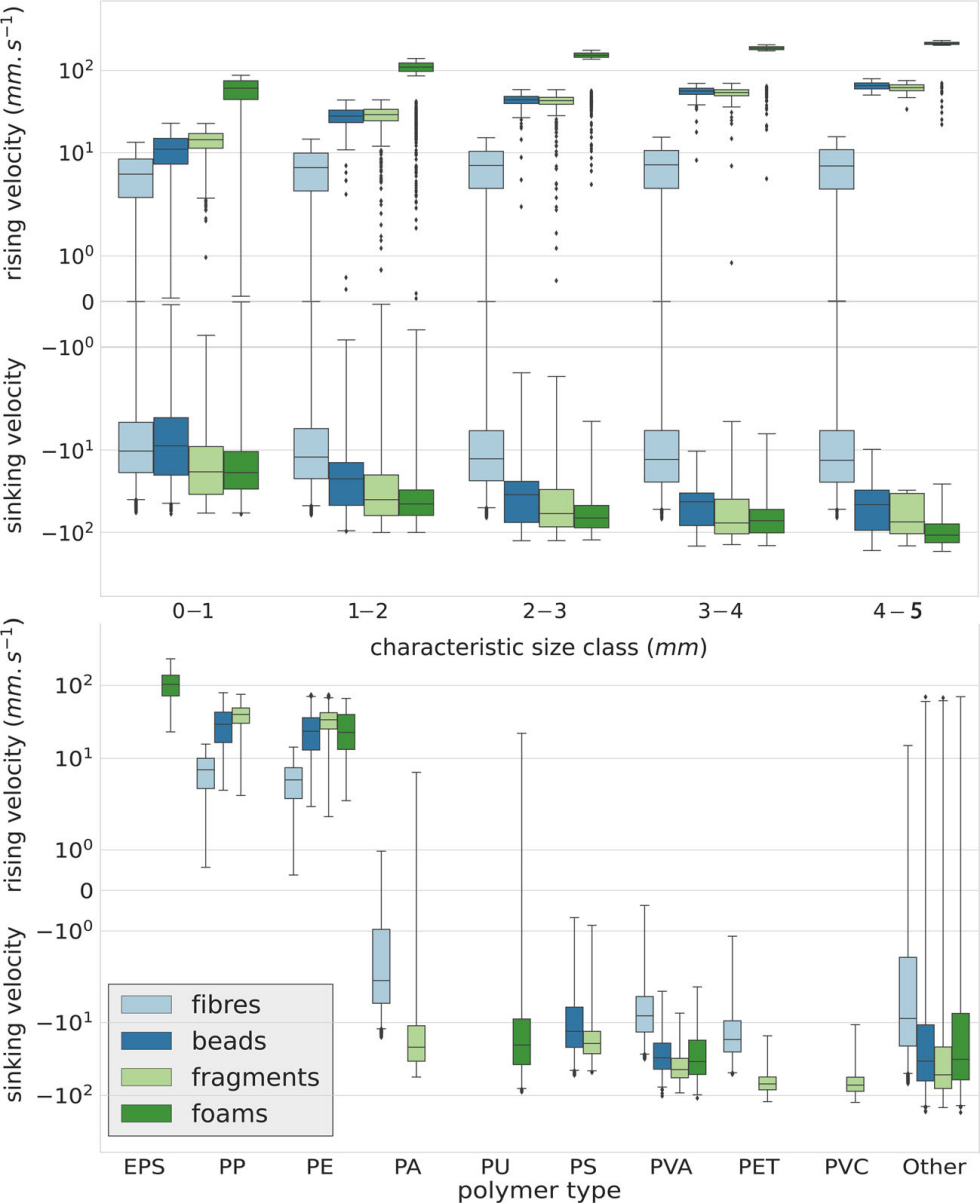
Distribution of rising and sinking vertical velocities associated with MPs discharged by the Mediterranean rivers into the sea, according to their physical properties

Based on the previously described set of physical properties (Fig. 3), our simulation counted 65% of floating MPs and 35% of dense MPs. The simulated ranges of rising and sinking velocities calculated from Eqs. 2 and 3 are displayed in Fig. 4. The average rising velocity was 0.006 $m \ s^{-1}$ for floating fibers, 0.035 $m \ s^{-1}$ for floating fragments, 0.027 $m \ s^{-1}$ for floating beads, and 0.1 $m \ s^{-1}$ for floating foams. The average sinking velocity was $-$0.014 $m \ s^{-1}$ for dense fibers, $-$0.049 $m \ s^{-1}$ for dense fragments, $-$0.031 $m \ s^{-1}$ for dense beads, and $-$0.043 $m \ s^{-1}$ for dense foams (Fig. 4 and Table 3). Whatever the size class, fibers generally had a lower rising and sinking velocity than other MP shapes, favoring their mixing in the water column by ocean dynamics. Foams, on the other hand, were mainly made of low-density EPS and therefore had high rising velocities, making them float on the surface, but some had also high sinking velocities, due to a PU or PVA composition that made them sink rapidly.

**Table 3:** Extreme values (min, max) and mean values for dense MPs (*D*.*MP* with sinking (negative) velocities) and floating MPs (*F*.*MP* with rising (positive) velocities)

	$w_s (\text {m.s}^{-1})$				Density (kg.m$^{-3}$)				$d_{eq}$ (mm)				Length (mm)			
	Min	Max	Mean	Mean	Min	Max	Mean	Mean	Min	Max	Mean	Mean	Min	Max	Mean	Mean
			D. MP	F. MP			D. MP	F. MP			D. MP	F. MP			D. MP	F. MP
All MP	$-$0.17	0.23	$-$0.016	0.031	100	1600	1275	868	0.03	5	0.37	1.39	0.3	5	1.50	1.54
Fibres	$-$0.07	0.016	$-$0.014	0.006	900	1600	1280	915	0.03	0.5	0.26	0.27	0.3	5	1.50	1.51
Fragments	$-$0.15	0.075	$-$0.049	0.035	900	1600	1230	930	0.25	5	1.92	1.93	0.3	5	1.52	1.56
Beads	$-$0.17	0.079	$-$0.031	0.027	900	1600	1180	930	0.30	5	1.53	1.50	0.3	5	1.53	1.50
Foams	$-$0.17	0.23	$-$0.043	0.10	10	1600	1270	70	0.30	5	1.47	1.51	0.3	5	1.47	1.51

$w_s$ is the MP specific vertical velocity, and $d_{eq}$ is the MP characteristic size (see Eqs. 2, 3)

Figure 4 shows that size, shape, and density all had a significant effect on the vertical movement of MPs. For the same size class, shape, or polymer type, rising and sinking velocities can vary over 3 to 5 orders of magnitude, with overlapping intervals, making it difficult to link the multitude of dynamic behaviors with specific characteristics when these physical properties are mixed together. Fiber sinking and rising velocities vary from $-$0.07 to $-10^{-5} \ m \ s^{-1}$ and $-10^{-5}$ to 0.016 $m \ s^{-1}$, bead and fragment vertical velocities from $-$0.2 to $-10^{-3} \ m \ s^{-1}$ and $-10^{-3}$ to 0.08 $m \ s^{-1}$, and EPS foam rising velocities from 0.03 to 0.23 $m \ s^{-1}$ (Table 3).

**Fig. 5:**
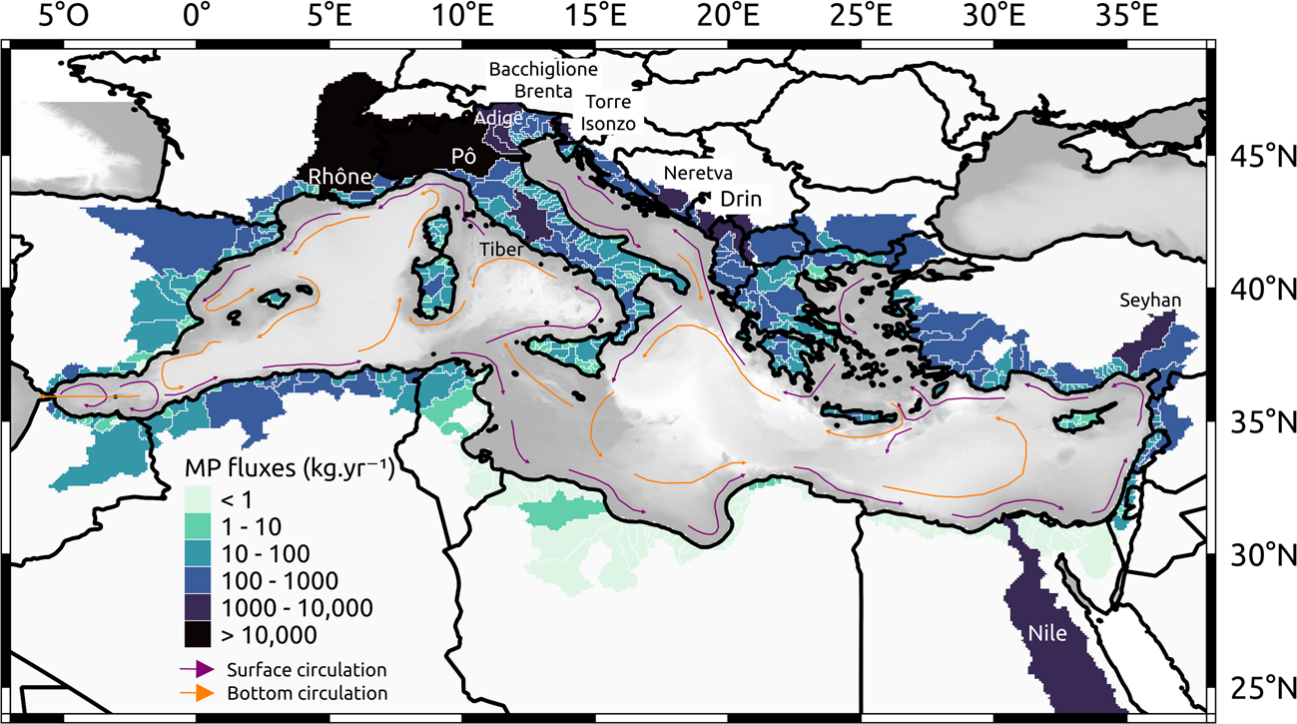
Mean annual MP fluxes discharged by river basins into the Mediterranean Sea, based on the empirical model of Weiss et al. (2021) and the river basin delineation of Sadaoui et al. (2018)

Our semi-random configuration of turbulent vertical diffusion based on kinetic energy caused floating MPs to mix at depths of tens to hundreds of meters under particularly turbulent conditions. We observed in the simulation a rising velocity threshold for vertical mixing in the surface layer: above 0.06 $m \ s^{-1}$ floating MPs were no longer affected by turbulence and drift strictly on the surface (it concerns EPS foams and a very small proportion of light $>2mm$ fragments and beads), and below 0.06 $m \ s^{-1}$ MPs can be mixed down to 200 *m* depth (as $<1mm$ PE or PP fragments and PE, PP, or PA fibers of all sizes). Rising velocities below $10^{-3} \ m \ s^{-1}$, mainly observed for short and light fibers, can reach 1000 *m* depth following the 3D general circulation. For dense MPs, the closer their sinking velocity was to 0 ($-10^{-3}$ to $-10^{-5} \ m \ s^{-1}$), the longer were their trajectories (as PA, PVA fibers or small PS, PU fragments). MPs with high sinking velocities ($< -0.05 \ m \ s^{-1}$) reached the seafloor quickly and remained mainly on continental shelves at shallow depths (as $>1mm$ PET or PVC fragments and beads).

### Modeled river inputs

The spatial distribution of river MP fluxes (Fig. 5) shows that the river basins on the northern shore of the Mediterranean clearly dominated the fluxes compared to the southern shore, reflecting their correlation with water runoff. The runoff was globally 1 to 2 orders of magnitude greater in the North, with averaged values from 100 to 1000 $mm \ year^{-1}$, than in the South, with averaged values from 10 to 100 $mm \ year^{-1}$. The resulting specific MP fluxes ranged from 0.1 to 50 $g \ km^{-2} year^{-1}$ in the South versus 10 to 500 $g \ km^{-2} year^{-1}$ in the North.

According to the model, the four largest contributors to MP pollution were the Rhone with $\sim$11.9 $t \ year^{-1}$ (15% of total inputs), the Po with $\sim$10.6 $t \ year^{-1}$ (13%), the Drin with $\sim$5.5 $t \ year^{-1}$ (7%), and the Nile with $\sim$5.2 $t \ year^{-1}$ (6.5%). The Rhone and the Po are the two main rivers supplying freshwater to the Mediterranean with average water flows of 1720 $m^{3} s^{-1}$ and 1570 $m^{3} s^{-1}$ respectively, i.e., 26% of the total freshwater discharge to the basin (Ludwig et al. 2009). With a smaller basin area and the fifth largest water discharge after the Nile and the Ebro, the Drin, flowing through Albania and Montenegro to the Adriatic Sea, had the third largest MP flux. With its 3 million $km^{2}$, the Nile Basin is by far the largest river basin in the Mediterranean region but the construction of dams and massive water extractions for irrigation have drastically decreased its freshwater input to the Mediterranean Sea (Ludwig et al. 2009; Sadaoui et al. 2018). Although these figures are highly debated, the Nile’s flow would drop to only 1740–1900 $m^{3} s^{-1}$ downstream of the Aswan Dam (Schroeder et al. 2012), resulting in a flow at the mouth of the order of 475 $m^{3} s^{-1}$.

**Fig. 6:**
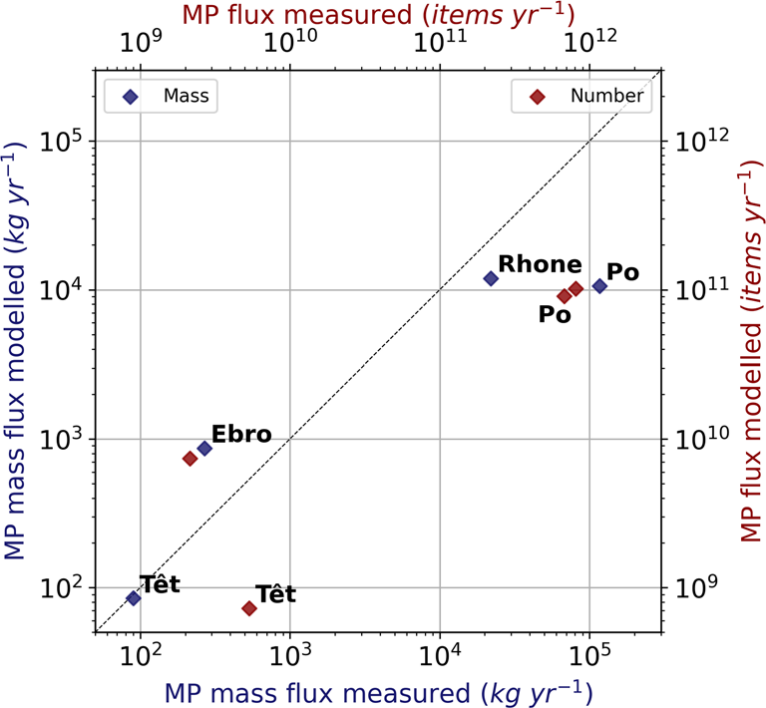
Comparison between measured and modeled MP fluxes in mass (blue) and in number (red)

The next important contributions were the basin of Bacchiglione and Brenta in Italy with 1.9 $t \ year^{-1}$ (2.4% of total inputs), the Neretva River in Croatia with 1.4 $t \ year^{-1}$ (1.8%), the Torre and Isonzo in Italy with 1.4 $t \ year^{-1}$ (1.7%), the Adige in Italy with 1.3 $t \ year^{-1}$ (1.6%), the Seyhan in southern Turkey with 1.1 $t \ year^{-1}$ (1.4%), and the Tiber in western Italy with 1.0 $t \ year^{-1}$ (1.3%). These ten river basins were responsible for 52% of the riverine MP fluxes to the Mediterranean (Fig. 5).

**Fig. 7:**
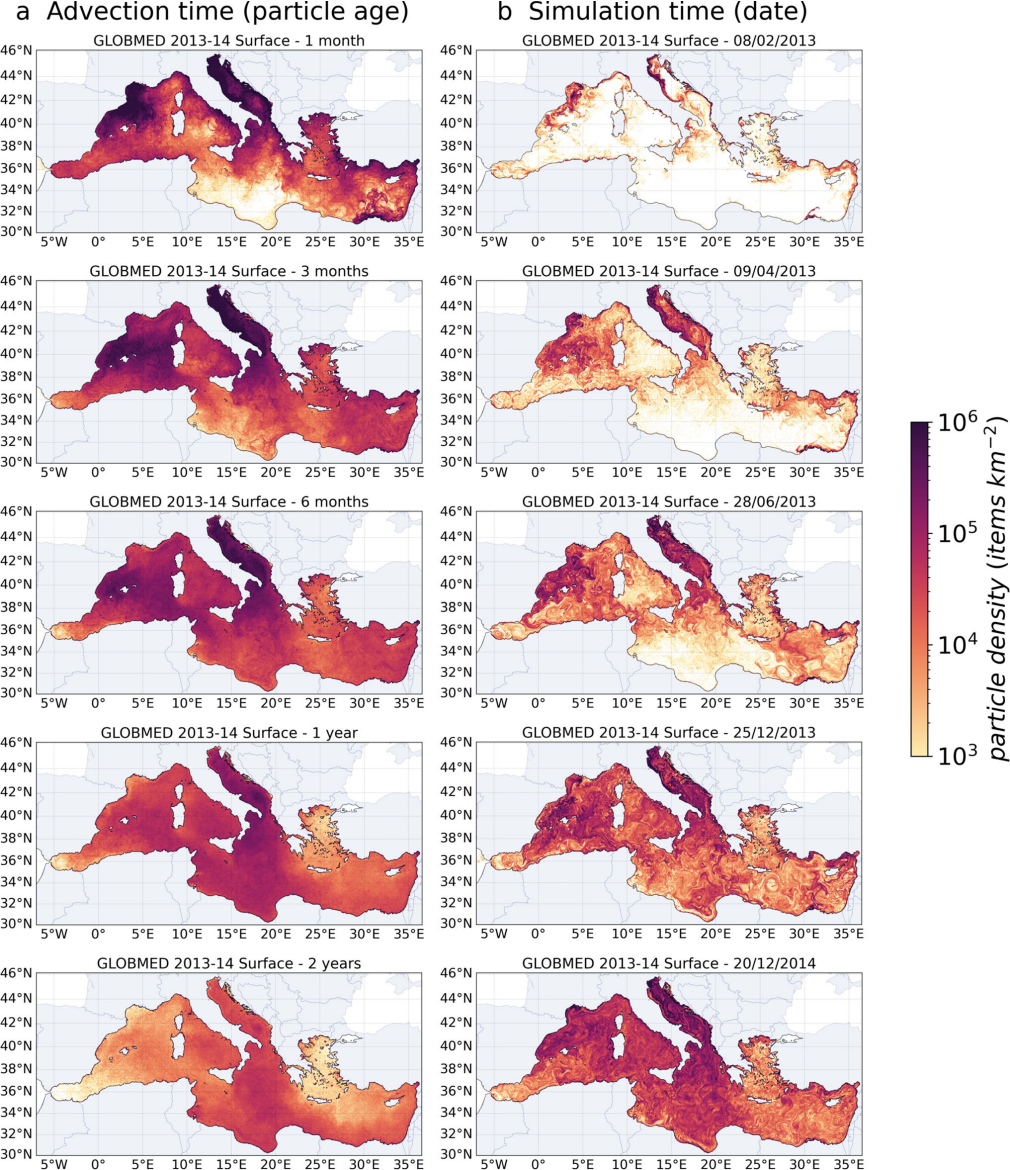
Concentrations of floating MPs in the surface layer **a** for different advection times, i.e., all the MPs have the same drifting time on each map (ages 1 month, 3 months, 6 months, 1 year, and 2 years), regardless of their release date from early 2013 to late 2014, and **b** for different simulation times, i.e., chronological snapshots at a given date with a continuous MP release over the 2-year simulation (from early 2013 to late 2014)

**Fig. 8:**
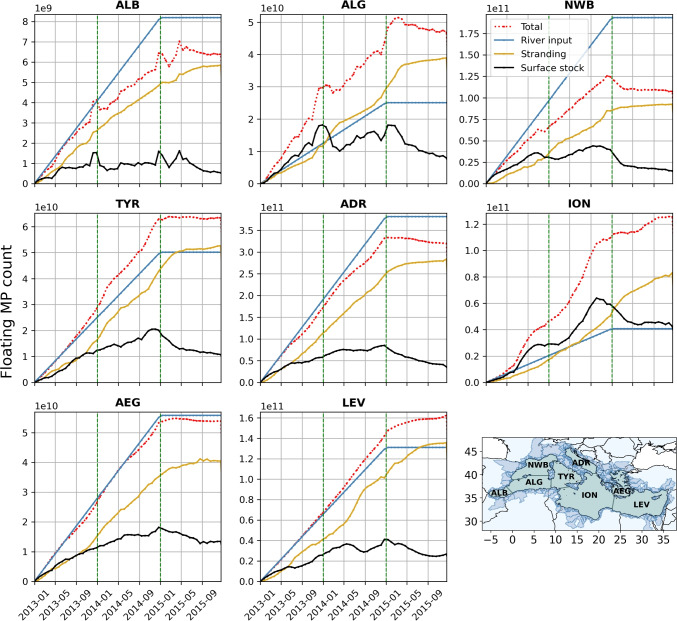
Temporal variation of the floating MP count in Mediterranean sub-basins. The total particle count (red curve) corresponds to the stranding (yellow) plus surface stocks (black). The blue curve represents cumulative river inputs. ALB Alboran Sea, ALG Algerian Basin, NWB North-Western Basin, TYR Tyrrhenian Sea, ION Ionian Basin, ADR Adriatic Sea, AEG Aegean Sea, LEV Levantine Basin

Figure 6 shows the comparison between measured and modeled MP fluxes for four monitored Mediterranean rivers. For the Têt, a small coastal river in the South of France, the modeled mass fluxes corresponded well to the observations from Constant et al. (2020). For the Ebro (Simon-Sánchez et al. 2019) and the Rhone (Constant et al. 2020), whose basins are larger and more populated, the orders of magnitude were respected, while for the Po (van der Wal et al. 2015), the model underestimated the fluxes by one order of magnitude. Our flux for the Rhone (12 $t \ year^{-1}$) was between the estimated flux during normal conditions, i.e., 5.92 $t \ year^{-1}$, and the flux including samples during flooding, i.e., 22 $t \ year^{-1}$ (Constant et al. 2020). Concerning the fluxes expressed in the number of MPs, except for the Ebro, the conversions also tended to underestimate the fluxes. The normalized root mean square error (NRMSE) is equal to 46% for the mass fluxes and 57% for the fluxes in the number of MPs considering those four rivers.

### Dispersion in the surface layer

#### Temporal tracking of floating MPs

Figure 7 shows the evolution of the floating MP concentrations in the surface layer over time, illustrating the sub-basin exchanges between the Alboran Sea (ALB), Algerian Basin (ALG), North-Western Basin (NWB), Tyrrhenian Sea (TYR), Ionian Basin (ION), Adriatic Sea (ADR), Aegean Sea (AEG), and Levantine Basin (LEV) (see Fig. 8). The 1-month and 3-month maps of the first panel (Fig. 7a) show that the NWB and ADR basins rapidly dispersed their river inputs first to the central and then to the southern sub-basins. In the ADR, which alone may have received nearly 40% of the total river inputs, the transfers occurred from the major river sources in the north to the cyclonic gyre in the south, which gradually accumulated MPs (panels Fig. 7b). After a 2-year advection time (last panel Fig. 7a), this transient accumulation zone still showed among the highest surface concentrations. Despite the narrowing of the ADR at the Otranto Strait limiting surface exchanges, part of the MPs retained in its southern gyre reached the Gulf of Taranto to the north of the ION (first pannels on Fig. 7b).

Figure 7a also shows that the concentrations at the surface of the Gulf of Lion decreased exponentially when particle age increased: by one order of magnitude between 1 and 3 months, by 2 orders of magnitude after 6 months, and up to 3 orders of magnitude after 1 year. The MP residence time at the surface of the Gulf of Lion was very short compared to the ADR. This rapid MP export favored a strong accumulation in the Balearic Sea and the North Balearic frontal zone (see the surface circulation Fig. 5). With continuous river inputs, the peak concentrations on the North-Balearic front were permanent (Fig. 7b), but the trapping efficiency did not exceed a year, due to a MP leakage towards the southeast (Fig. 7a).

The central ION exhibited a long-term MP accumulation with an overall increase in mean surface concentrations between the 1-month and the 1-year maps (Fig. 7a). After 2 years, surface concentrations in the ION were of the same order of magnitude as those in the ADR while its sources were almost ten times lower (Table 3). Concentrations at the surface of the LEV tended to be homogenized by its cyclonic circulation (Fig. 5), with a long-term accumulation rather located in its southern and eastern parts. This homogenization was also observed in the TYR with higher concentration in the center of the sub-basin, supplied by the strong fluxes of the Tiber. The strait between Corsica and Italy and the seasonality of the East Corsican Current limited the MP outflow to the north and the cyclonic circulation in the sub-basin favored stranding rather than their exit to the south.

In the AEG, the flow of desalinated and light water from the Black Sea produced a cyclonic surface circulation along the northern coast and then along the eastern coast of Greece, constituting a barrier to MP export off the coasts. The numerous islands, indented coastline, and deep bays also increased the retention of MPs in coastal areas. The absence of discharges into the Marmara Sea through the Bosphorus Strait can have impacted the distribution of particles in the AEG.

At the Strait of Gibraltar, as the Atlantic waters enter at the surface and the dense Mediterranean waters exit at depth, the leakage of floating MPs to the Atlantic Ocean was negligible. Only about 1 million floating MPs reached the Atlantic in 2 years of simulation (annual leakage of 0.00015% of the riverine sources). Although very few floating MPs left the Mediterranean Basin, the strong decrease in surface MP concentrations (by a factor of 5 between the 1-month and 2-year maps, Fig. 7a) indicated important stranding. This was confirmed by the fact that, between the 1-year and 2-year concentration maps (Fig. 7b), riverine inputs were multiplied by 2, while surface concentrations were not.

#### MP count variation in sub-basins

Annual stranding rates reached almost 90% for floating MPs and 46% for dense MPs in the Mediterranean Basin when all particles had completed 2 years of drift. In the following, we assume that the time variations in the number of particles in the sub-basins are correlated with the prevailing circulation and therefore with the total inputs and outputs of the sub-basins.

With a 10% leaking rate of floating MPs, NWB is the sub-basin with the strongest net export to the rest of the Mediterranean, i.e., 43 billion MPs $year^{-1}$ or 45% of its annual river inputs (difference between blue and red curves in Fig. 8, NWB). Following the circulation in the basin (Figs. 5, 7), NWB export would contribute to a possible net increase of 10 billion floating MPs $year^{-1}$ in the ALG, i.e., 77% of its river sources. This NWB-to-ALG export appears to occur preferentially during winter, as evidenced by the observed increase in surface stocks of ALG and decrease in surface stocks of NWB between December and January (see Fig. 8, ALG-NWB). The ALG was a transit basin on the path of MPs towards the TYR which represented a net annual increase of 7 billion floating MPs $year^{-1}$ ($+$28% of its river inputs) and towards the ION. The amount of floating MPs entering NWB should be low compared to its own sources and exports due to the limiting MP export from TYR to the Ligurian Sea.

In the eastern part of the Mediterranean, the outflow from ADR at the Otranto Strait is generally stronger than the inflow, resulting in a net decrease of 31 billion floating MPs $year^{-1}$ in ADR, i.e., 16% of its annual river inputs (red curve on Fig. 8, ADR). These net exports from ADR combined with those from the western basin resulted in a net increase of about 40 billion floating MPs $year^{-1}$ in ION, preferentially occurring in summer between June and September. The ION is the only sub-basin for which the net particle count variation was twice as high as its annual river inputs ($+$197%). Thus, although its river inputs were low, ION constituted the main accumulation basin of the Mediterranean that encounters a $+$9% net annual increase of the total MP count in the Mediterranean Basin.

**Fig. 9:**
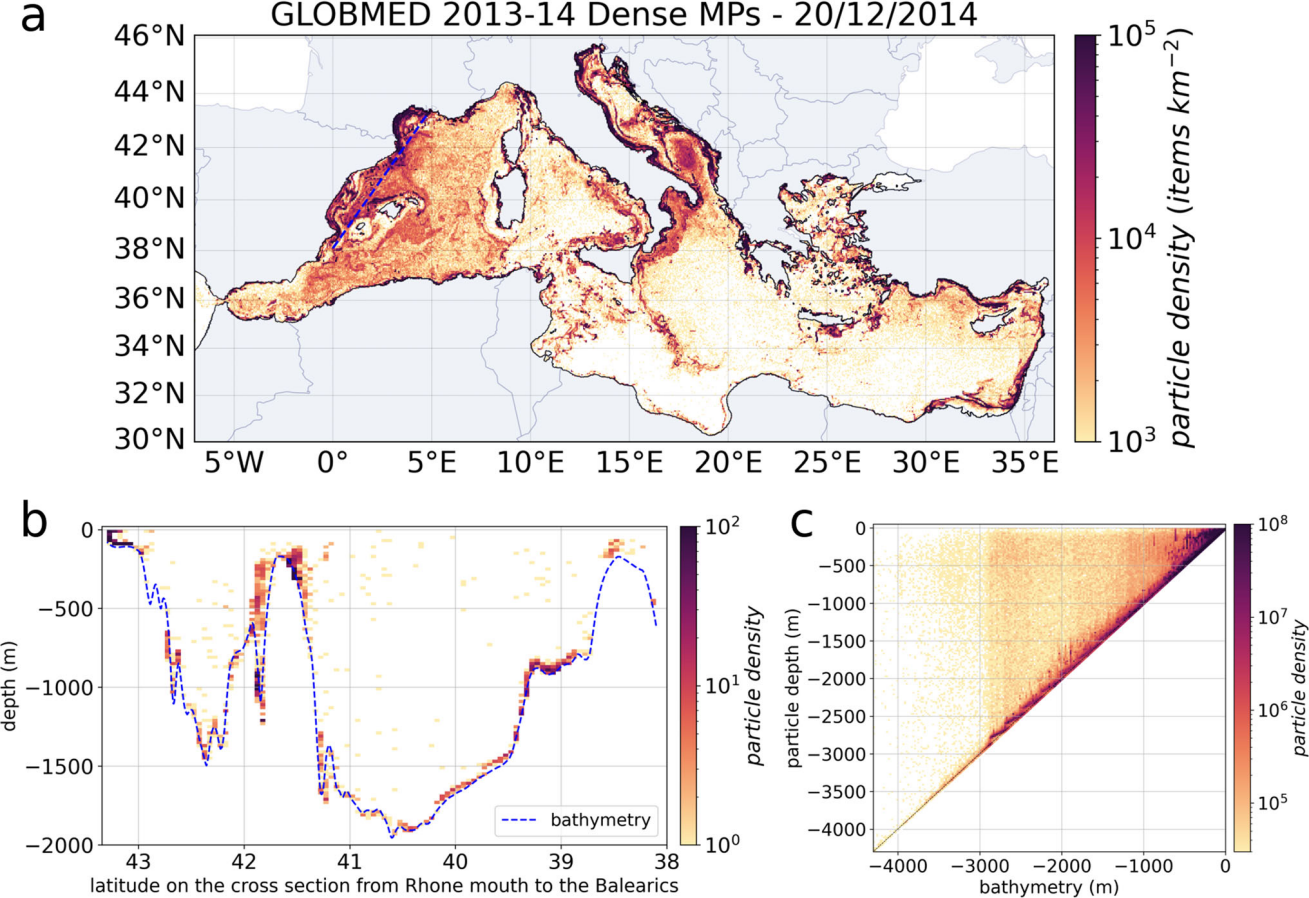
Sinking MP concentrations after 2 years of simulation **a** in the bottom layer, **b** along the vertical blue section from the Rhone River mouth to the Balearic’s, **c** as a function of depth for the whole Mediterranean Sea

Some MPs from ION would be exported to LEV, which faced a net increase of 19 billion floating MPs $year^{-1}$, i.e., 29% of its river sources and 4.4% of the total MP concentration in the basin (Fig. 8, LEV). The cyclonic surface circulation that closes the LEV south of Crete reduced the export of MPs to adjacent sub-basins. To the northeast, AEG particle count decreases only 3% of its river inputs, i.e., less than 1 billion floating MPs $year^{-1}$. The export from AEG occurred preferentially in late summer to autumn (see red curve on Fig. 8, AEG).

### Distribution at depth

The dispersion of sinking MPs was much slower due to deep currents, which can be one order of magnitude slower than surface currents, resulting in smaller travel distances. As a result, exchanges of dense MPs between sub-basins were minimal: the MP concentrations after 2 years were very close to the MP fluxes released in each sub-basin. Dense MPs accumulated along the coast in the shallow domain and along the continental slopes, as can be seen in the Gulf of Lion, the Balearic Sea, the ION, and the southern LEV (Fig. 9a). As the dense MPs transit at depth, they were not sensitive to wind-induced currents or Stokes drift and therefore preferentially follow the general circulation. Figure 9a highlights the effect of the cyclonic circulation of intermediate and deep water that distributed the MPs in the western basin (see circulation in Fig. 5). About 340 million sinking MPs exited through the strait of Gibraltar to the Atlantic, representing 240 times the surface outflow.

Moreover, dense MPs tend to sink rapidly. Looking at the section from the Rhone River mouth to the Balearic Sea (Fig. 9b), the highest amounts of dense MPs were found on continental shelves but also in canyons such as the ones that exit the Gulf of Lion. However, after 2 years, we still found millions of sinking MPs suspended throughout the water column. On average, 2 orders of magnitude separated the MP concentrations in the water column from the ones close to the seafloor (Fig. 9c).

Half (48%) of the dense MPs emitted by rivers were located between 50 and 500 *m* depth, and the majority remained in the coastal zone. All types of dense MPs were found on these shelves, but the MPs that reached rapidly the bottom near their source were the ones with the highest sinking velocities, i.e., the largest MPs in size, mainly composed of PET and PVC. Maximum concentrations on continental shelves reached 150 to 200 million MPs $km^{-2}$ (Fig. 9a). The dense MPs that went further offshore ended up in the water column in the center of the sub-basins. They had low sinking velocities of the order of $10^{-5}$ to $10^{-4} \ m \ s^{-1}$ and corresponded mainly to fine PA (nylon) and PVA (acrylic) fibers with densities between 1.02 and 1.1 $g \ cm^{-3}$ as well as to the smallest ($<< 1 mm$) PS or PU fragments or beads (Fig. 4).

**Fig. 10:**
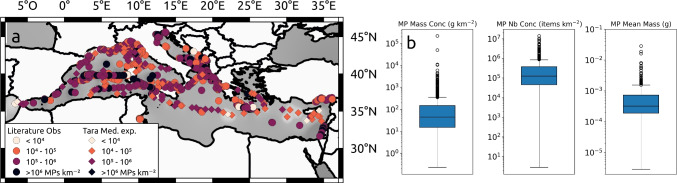
**a** MP concentrations observed at the Mediterranean Sea surface (in MPs $km^{-2}$) compiled from literature reviews and **b** variability of the floating MP concentrations in mass and in number of MPs with the variability of the mean mass of each MP particles sampled by plankton nets

Only about 3% of the dense MPs (7 billion) reached depths between 500 and 1000 *m* after 2 years. It is about the same fraction that reached depths greater than 1000 m in the center of the sub-basins, especially in the western part. The maximum concentrations modeled after 2 years below 500 *m* depth were between 20 and 25 million MPs $km^{-2}$. This observation was related to the type of sources considered here exclusively located on the coastline.

In the deepest water layer below 1000 *m*, relatively high concentrations were found in the Algerian basin where MPs were transported by dense water masses formed in the Gulf of Lion convection zone. These MPs did not reach the Atlantic because they remained below the Gibraltar threshold. Information on the MP distribution in the water column and seafloor remains rare as sampling those compartments is still complex even if plastic accumulation has already been observed in the shelves and canyons (Kane and Clare 2019).

### General comparison with observations

We compiled around 600 measurements of floating MP concentrations (Fig. 10), based on the review of Mansui et al. (2020) and the Tara Mediterranean expedition (Pedrotti et al. 2022), to compare with our simulation. Figure 10b illustrates the significant spatial and temporal variability that can exist in surface MP concentrations, depending on hydrodynamic conditions (currents, winds, turbulence, surface mixing, Kukulka et al., 2012), seasonality of sources or sampling protocols. Observations at the same location can vary within 2 orders of magnitude (for example, transects west of Sardinia in Fig. 10a). However, the spatial and even more temporal resolution of the observations is low, and key areas such as ION, LEV, and AEG are still poorly covered.

We compared concentrations at different simulation time steps (6 months, 1 year, 2 years) with observations based on the coordinates of the measurements, but no correlations were found. Nevertheless, the orders of magnitude obtained by our model are close to the sampled concentrations (comparison between Figs. 7 and 10a). Concentrations observed at the surface ranged from 2 to 14 million MPs $km^{-2}$ and from 0.2 to 220,000 $g \ km^{-2}$ (Fig. 10b). The median values are 124,000 MPs $km^{-2}$ and 45 $g \ km^{-2}$ respectively. Our modeled concentrations offshore ranged from 375 MPs $km^{-2}$ to 5 million MPs $km^{-2}$, representing 0.04 to 584 $g \ km^{-2}$, after 2 years of simulation (Fig. 7b). The median concentration modeled in the surface layer was about 18,000 MPs $km^{-2}$, i.e., 2 $g \ km^{-2}$ based on our conversion factors. Multiplying these median values by the total sea surface (2,501,000 $km^{-2}$), we obtained an observed average of 310 billion MPs, i.e., about 112 *t*, and a modeled average of 46 billion MPs, i.e., about 5.4 *t* at the Mediterranean surface.

The boxplot to the right of Fig. 10b shows the variability of the MP mean mass in the samples, varying from 2.9 $\mu g$ to 0.03 *g* with a median mass of 0.322 *mg*, slightly higher than the average mass obtained for MPs in river samples equal to 0.745 $\mu g$ for fibers and 0.233 *mg* for other MP shapes (Weiss et al. 2021). This suggests preferential sequestration of the smallest MPs from the sea surface, in line with our model, where vertical mixing affected the lightest floating MPs, with rising velocities $< 0.06 \ m \ s^{-1}$. They consequently may sink in the water column down to 200 *m* depth (or sometimes even 1000 *m* for rising velocities $< 10^{-3} m \ s^{-1}$).

Regionally, the observations from Fig. 10 show the highest concentrations in the coastal zone, mostly near river mouths, which are highlighted as major contributors by our river input model. For example, waters east of the LEV Basin are under the influence of the Nile and Seyhan rivers, high concentrations in the northern ADR were sampled close to the Po, Adige, Bacchiglione, Brenta, Torre, and Isonzo rivers, and inputs from the Rhone and Ebro rivers may explain the hotpots around the Balearic Islands. Offshore, Suaria et al. (2016) report very high concentrations of more than 1 million MPs $km^{-2}$ in the southern ADR gyre, identified as a trapping zone in our simulation with concentrations between 0.1 and 5 million MPs $km^{-2}$ (Fig. 7).

## Discussion

### MP sources

Our quantitative distribution of modeled sources along the coast was very different from other Lagrangian studies in the Mediterranean, mostly based on the MPW-derived riverine inputs of Lebreton et al. (2017). The inputs we used corrected methodological errors made in previous estimates (demonstrated and discussed by Weiss et al., 2021) and reassessed global and Mediterranean-scale fluxes 2 to 3 orders of magnitude lower (80 $t \ year^{-1}$ or 682 billion MPs $year^{-1}$ here). The most conservative estimates to date come from Kaandorp et al. (2020) (2100 to 3400 $t \ year^{-1}$) for the total fraction of plastics originating from three different types of sources to the Mediterranean Sea (rivers but also coastal populations and fisheries that were not accounted in our study). They predicted a contribution from rivers of only 32% (i.e., 672 to 1088 $t \ year^{-1}$). Moreover, their method considers a wider range of particle sizes (micro $<5mm$ and macro $>5mm$) than ours (micro $<5mm$ only). Thus, our 80 $t \ year^{-1}$ of river MPs would correspond to a fraction of 7 to 12% of their estimates of total river inputs, which is a plausible assumption. It suggests that the estimates of the order of 100,000 $t \ year^{-1}$ widely used in the literature (Liubartseva et al. 2018; Soto-Navarro et al. 2020; Pedrotti et al. 2022; Baudena et al. 2022) are likely overestimated, as these studies do not match the order of magnitude of the concentrations observed.

Although the quantification of inputs is very different, we used almost the same number and location of seeding points as previous modeling studies (505 cities and 110 rivers by Liubartseva et al. (2018); 480 cities and 15 rivers by Soto-Navarro et al. (2020); 185 cities and 200 rivers by Pedrotti et al. (2022); 549 river mouths in our study), underlining the benefit of using a high-resolution river basin GIS. Following MPW-based river models, Kaandorp et al. (2020) do not even consider the Rhone as a main source while its contribution might be among the major ones based on observations (Constant et al. 2020). The use of a high-resolution GIS covering the entire continental area, in combination with flux estimates based on population and water runoff, permitted to take into account the contributions of all rivers and cities (including coastal cities) in the 549 simulated river sources. However, only the portion of the MP fluxes exported by urban areas towards the sea by water runoffs was included in those estimates (the weight of water flows in the parametric equation was stronger than the one of population Weiss et al. 2021). It should be noted that our MP flux estimates did not include all direct contributions from coastal activities, such as inputs from atmospheric deposits, coastal wastewater treatment plants, and human emissions on beaches or in harbors. Consequently, our sources are likely to be underestimated when compared to the total contributions of other studies or actual inputs. Moreover, the fragmentation of macroplastics as a source of MPs (Pedrotti et al. 2022) was not included either in the model. In fact, the fragmentation process seems to remain very slow occurring over decadal time and depending on a multitude of variables (Onink et al. 2022). It begins in the continental environment, on river banks and streams during their transfer, or directly on beaches. It is therefore very difficult to quantify the proportion of MPs derived from stranded macroplastics in comparison to other sources.

Contrary to MPW-based studies, our MP fluxes were 1 to 2 orders of magnitude lower in southern river basins than in northern river basins. For example, the Nile contributed in our study only for 6.5% of total inputs to the Mediterranean Sea, whereas the application of MPW-based models (Lebreton et al. 2017) led to an estimated contribution of 60–90% of the total inputs. This is in contradiction with the strong reduction in this river discharge because of dam retention and irrigation (Ludwig et al. 2009; Schroeder et al. 2012; Sadaoui et al. 2018). We noted a significant relationship between MP concentrations observed in the coastal zone and the importance of modeled river sources (section General comparison with observations), suggesting that river inputs could indeed be major sources that appear to be well estimated by our model. Nevertheless, the uncertainties related to the modeled river fluxes are still high because too few observations are available in Mediterranean rivers, particularly on the southern shore, and the river model only provided annual averages which do not express the seasonal and high-frequency variability of flows.

### MP characterization

#### Shape, size, and polymers

Considering 50% fibers at river mouths should be taken as a lower limit. When fibers are counted, they are by far the most dominant shape in plankton net samples (up to 90% according to Constant et al. 2020). Also, fragments (35%, Table 2) were often reported in large quantities in literature samples, likely derived from the degradation and fragmentation of macroplastics. Beads were less frequent (10%) probably related to their general lower buoyancy which keeps them under the sampled surface. Foams (5%), mostly made of EPS, were likely to degrade quickly in water.

**Fig. 11:**
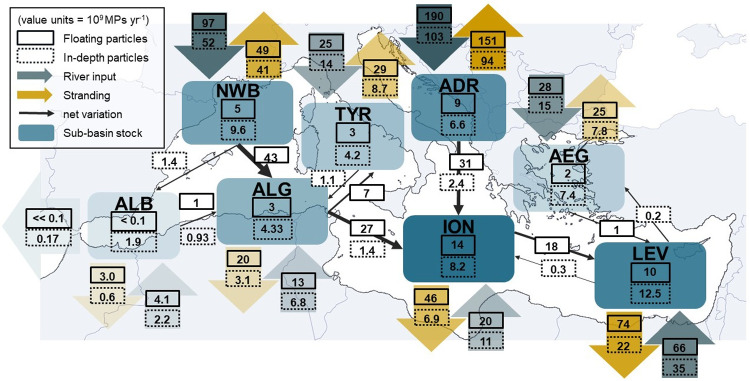
Balance scheme of annual particle count in the Mediterranean sub-basins. Black arrows indicate the estimated net fluxes between sub-basins in accordance with the prevailing circulation, and numbers in the regional boxes correspond to the MP count in the surface layer and at depths

Concerning the MP sizes, the distribution we averaged from literature data followed a trend observed in many other case studies in rivers like in the Yangtze river (Xiong et al. 2019), the Ebro river (Simon-Sánchez et al. 2019), or the Pasig river (Deocaris et al. 2019) as well as in the Mediterranean Sea (Cózar et al. 2015; Pedrotti et al. 2016).

In the same way, the predominance of PE and PP in our analysis of literature data (Fig. 3) was confirmed by many other studies like in global rivers (Xiong et al. 2019; Kataoka et al. 2019; Lahens et al. 2018; Faure et al. 2015; van der Wal et al. 2015) and in the Mediterranean Sea (Kedzierski et al. 2022). Their proportions were highly variable (30 to 80% for PE and 6 to 50% for PP—compared to 37% PE and 22% PP in observed distribution), with PE/PP ratio of between 1.6 and 6, showing the usual prevalence of PE in the environment. It also suggests that, despite degradation or biofouling affecting MP density, many of these PE and PP particles continue to float at the surface for a long time, the drift period depending on biological and physical activities in the region. High-density polymers like PS, PET, PVC, or PU can also be found but to a lesser extent (4 to 25% maximum) in river plankton net samples (Xiong et al. 2019; Kataoka et al. 2019; Lahens et al. 2018; Faure et al. 2015; van der Wal et al. 2015; Sadri and Thompson 2014) and only 3% PS in the Mediterranean Sea (Kedzierski et al. 2022). Although the density of the later polymers is higher than seawater, their presence in surface samples is generally explained by the turbulent flow that mixes the water column in rivers and coastal waters. The additives and chemical treatments applied to polymers can also greatly alter their intrinsic properties and buoyancy.

#### Vertical velocities

As for sediments, MP rising and sinking velocities are key variables to understand suspension, deposition, mixing, and exchange processes. Our simulated velocities based on sampled properties and semi-empirical equations were within the ranges observed in lab experiments (Fig. 4, Table 3, Eqs. 2 and 3). Our average sinking velocity for fragments was equal to $-$0.049 $m \ s^{-1}$, which was included in the experimental interval from $-$0.097 to $-$0.014 $m \ s^{-1}$ (mean of $-$0.060 $m \ s^{-1}$) for high-density polymers ( 1130 $kg \ m^{-3}$) from Khatmullina and Isachenko (2017). Our average sinking velocities for beads ($-$0.031 $m \ s^{-1}$) and fibers ($-$0.014 $m \ s^{-1}$) were also close to the experimental values from Khatmullina and Isachenko (2017) equal $-$0.06 $m \ s^{-1}$ for beads and $-$0.016 $m \ s^{-1}$ for fibers. In their modeling study, Baudena et al. (2023) considered a maximum sinking velocity of 7.8 $m day^{-1}$, i.e., 9 $10^{-5} \ m \ s^{-1}$, which is 2 to 3 orders of magnitude slower than the velocities calculated in our observation-based study. Our average rising velocity for fragments (0.035 $m \ s^{-1}$) was higher than the experimental mean rising velocity from Kooi et al. (2016), ranging from 0.009 to 0.019 $m \ s^{-1}$. The difference can be due to the calculation of rising velocities with the same formula as sinking velocities, whereas some observations showed that, for the same difference between the MP density and surrounding water density, rising velocities were slightly lower than sinking velocities related to the ratio between the forces (weight and buoyancy) applied to the particle (Waldschläger and Schüttrumpf 2019). This discrepancy no longer existed with the formula we applied to fibers (0.006 $m \ s^{-1}$) which matched the experimental range between 0.006 and 0.008 $m \ s^{-1}$ by Kooi et al. (2016).

### MP fluxes in the basin

Here, we assume that the simulation has reached a quasi-stationary state of MP concentrations at the sea surface, as suggested by the quasi-stabilization of surface stock curves after 2 years of drift in the majority of sub-basins (see Fig. 8). By dividing the MP floating stocks in the regional boxes by the floating stranding losses (represented by the yellow arrows in Fig. 11) plus the net outputs (outgoing black arrows at the interfaces with neighboring boxes), it is possible to estimate a maximum average residence time of floating particles at the sea surface. This is approximately equivalent to dividing floating stocks by floating river inputs (blue arrows) plus the net inputs (incoming black arrows at the interfaces with neighboring boxes). The resulting time values represent maximum residence times, and actual residence times may be shorter.

ALB was the sub-basin with the smallest average residence time of floating MPs in the surface layer, about 9 days, then followed NWB, ALG, and ADR, with average residence times of about 3 weeks. AEG, TYR, and LEV yielded average residence times of 4, 5, and 7 weeks, respectively. Finally, ION is the sub-basin with the longest average residence times, reaching about 9 to 11 weeks. These average residence times were relatively short in such a semi-enclosed basin as the Mediterranean, as stranding can occur very quickly after MPs have entered the marine environment, while a smaller number of particles can drift for several years. In the same order of magnitude, Liubartseva et al. (2018) calculated average half-life times (when only 50% of the initial release remains at the sea surface) of about 7 days for floating MPs from coastal sources such as rivers and 80 days for floating MPs from marine sources. Here, we showed a wide range of average residence times, from a few days to 2.5 months, suggesting that a unique residence time cannot be applied to the whole Mediterranean since it depends on the sub-basins’ size and dynamics.

These average residence times may be long enough in the Mediterranean to allow living organisms to colonize a certain portion of floating plastic stocks, leading to exchanges between surface and deep stocks through changes in particle density. Some MPs made of PE would begin to sink after only 6 weeks of incubation in coastal waters with high biological activity Kaiser et al. (2017) while other PE fibers could start sinking after 6–8 months (Chubarenko et al. 2016). Thus, considering MP leakage from shipping lanes and fishing activities, as well as the change in the buoyancy of biofouled floating MPs, would have resulted in increased amounts of MPs in the center of the sub-basins with depths greater than 1000 *m* (Baudena et al. 2023).

### Basin scale budget

#### Stocks

To sum up our modeling scenario, rivers discharged 443 billion floating MPs $year^{-1}$ and 239 billion dense MPs $year^{-1}$ to the Mediterranean Sea. Total stranding reached 397 billion floating MPs $year^{-1}$ (almost 90% of floating inputs) and 110 billion dense MPs $year^{-1}$ (46% of dense inputs). Exchanges with the Atlantic were negligible. Considering this simplified MP cycle, the Mediterranean would accumulate offshore in average 46 billion floating MPs $year^{-1}$ in the surface layer and 54.8 billion sinking MPs $year^{-1}$ at depth (derived from Figs. 7b and 8).

This scenario failed to reproduce the average floating stocks observed, estimated at 310 billion MPs at the Mediterranean surface (Fig. 10). Evidently, observed accumulation is the result of more than 50 years of pollution, but monitoring at sea may show no clear increase in plastic quantities (Galgani et al. 2021). It suggests that there may exist a balance between sources and sinks in the marine environment. Thus, we assumed that the observed and modeled stocks should approximate a steady state at the Mediterranean surface in order to establish a budget for floating MPs in the basin. The assumption of a steady state after 2 years in our simulation can be justified, on the one hand, because 2 years of advection were sufficient to complete the transfer of floating MPs from western to eastern sub-basins (Fig. 7a), and on the other hand, because the surface concentrations in Fig. 7b with continuous daily river inputs change little between the 1-year and 2-year maps (10% increase while inputs doubled), suggesting that floating concentrations may stabilized (see surface stock curves on Fig. 8).

The most polluted regions in terms of river emissions were the ADR and the NWB. The ION constituted the main long-term accumulation sub-basin, as well as the southeastern LEV, with longer time transfers, about 2 years. The same accumulation sub-basins were suggested by the 18-year simulation of Macias et al. (2019). It would therefore be interesting to examine in sea samples whether the MPs in these basins are characterized by increased aging (i.e., increased degradation or biofouling rate) reflecting longer surface drift, although this is naturally difficult to determine since aging already starts within the river basins, where transfer time depends on basin scale and characteristics.

**Fig. 12:**
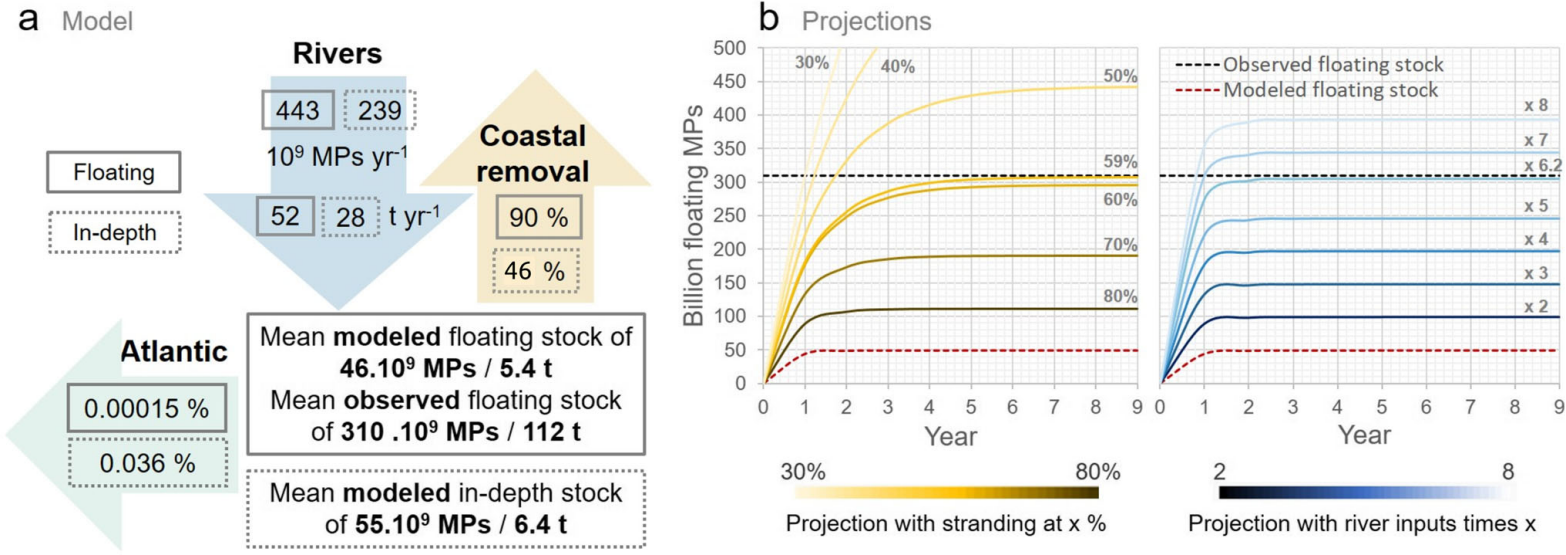
**a** Annual basin scale budget for the Mediterranean based on the modeled scenario and **b** projections of floating stocks based on several configurations of river inputs and stranding rates

#### Projections

We summarized our scenario with a 1D box model (Fig. 12a) and extended the statistics obtained from the 2-year simulation to a projected decade (Fig. 12b). Assuming that there is an observed surface steady state of about 310 billion floating MPs, our scenario either underestimated the sources or overestimated the sinks, or more likely both combined. By assuming only an underestimation of the sources on the one hand, sensitivity analyses based on our box model indicated that sources must be multiplied by about 6 (reaching approximately 2800 billion floating MPs $year^{-1}$ and 320 $t \ year^{-1}$) to reproduce an average stock that fitted to observations (right plot in Fig. 12b). This reflects the inherent uncertainties associated with the modeled river input estimates, which are potentially underestimated (Fig. 6) and do not consider extreme factors such as floods for example. And above all, the discrepancy between observations and our river-based model can be attributed to a number of additional unaccounted-for sources. These include direct coastal inputs via atmospheric deposits, coastal wastewater treatment plants, leakages on beaches and harbors, macroplastic fragmentation, and marine inputs from shipping lanes and fishing activities. A total MP source at 320 $t \ year^{-1}$ would represent 9 to 15% of the total plastic inputs predicted by Kaandorp et al. (2020) (2100 to 3400 $t \ year^{-1}$). On the other hand, we also assume that our permanent stranding hypothesis, resulting in 90% coastal removal, might be too drastic, given that beaches are eroding sedimentary systems (Vousdoukas et al. 2020). In this way, the box model sensitivity analyses indicated that to reach the observed floating stock, stranding rates should be reduced at least to 59% of the inputs (left plot in Fig. 12b). This reduction can be justified by considering that certain steep coasts, such as cliffs, can not trap MPs and that wind, waves, and currents can remobilize stranded MPs to the open sea (Haarr et al. 2019). When the stranding rate decreased, a longer simulation time was potentially needed to reach the steady state (more than 4 years). Another important perspective is to make the modeled MP cycle more complex by considering new sinks. For example, Baudena et al. (2022) estimated that biofouling could cause about 12% of floating MPs to sink to deeper layers. In our study, we already assumed that 35% of river MPs sink rapidly in coastal environments due to their density, adding the potential 12% biofouling of our 65% floating MPs, i.e., 7.8%, we would obtain 42.8% of MPs trapped at depth near the sea bottom, which is in the range of 37 to 51% estimated by the inverse model of Kaandorp et al. (2020). Degradation, fragmentation, or ingestion are other sinks that can lead to the removal of floating MPs from the surface. The existence of these additional sinks supports the necessity to reduce stranding rates to keep a modeled steady state close to the observed stock.

#### Stranding constrain

Our results entirely met the conclusions of Kaandorp et al. (2020) that used an inverse modeling methodology. One of their key results was a re-evaluation of plastic sources to the Mediterranean, 2 orders of magnitude lower than previous MPW-based studies. We have to recall here that our modeling scenario on river inputs also reassessed global river fluxes 2 to 3 orders of magnitude below MPW models (Weiss et al. 2021). Based on this initialization and a permanent configuration of stranding, the projection of the 1D box model shows that decreasing coastal removal to at least 59% of the river inputs would enable the model to reproduce the observed MP floating stock at the Mediterranean surface (around 310 $10^{9}$ MPs). The reduction in strandings would be even more important if other sinks were to be taken into account in the process of particle sequestration. This stranding rate was within the 49–63% range obtained by Kaandorp et al. (2020), who limited stranding by introducing a second sink via sinking probabilities based on surface residence times derived from biofouling and degradation of floating MPs.

The majority of modeling studies had difficulties defining coherent stranding hypotheses since they resulted in extremely high stranding rates (ranging from 98.7% by Macias et al. 2019 to 87% by Baudena et al. 2022). This was mainly due to the limitations of the coarse ocean grid resolution: a regional approach in a kilometer resolution is not small enough to realistically resolve coastal dynamic processes, and conversely, a high-resolution beach-scale study does not enable conclusions regarding regional transfers. This highlights that the community should make progress on the probabilistic characterization of stranding in regional modeling studies, based on refined hydrodynamics but also on empirical results from beach monitoring.

## Conclusion

The low spatial and temporal coverage of observations made it difficult to properly validate the model hypothesis and results. We had this in common with all previous modeling studies focussing on the Mediterranean. In this work, we therefore analyzed the MP dispersion in the Mediterranean Basin applying a new empirical model of river inputs, in-depth characterization of Lagrangian particles, and high-performance physical simulation. This allowed to increase the reliability of the trajectories calculated and to extend the conclusions to a simplified source-to-sink budget on a basin scale. This study focused only on rivers as a source and the coast as a sink. It did not include direct MP inputs from coastal cities or leakage from marine traffic, nor did it consider other complex processes affecting marine plastic debris, such as resuspension, fragmentation, biofouling, sedimentation, or washing-off. Two main transient accumulation structures were highlighted for floating MPs, the North Balearic Front and the South Adriatic gyre, as well as two main accumulation sub-basins, the Ionian Sea and Southeastern Levantine Basin.

Initializing the dispersion model with our revised source scenario, which reevaluated global river inputs at values 2–3 orders of magnitude lower than previous studies based on more homogeneous data sets, reversed the river contributions between the northern and southern shores of the Mediterranean. The northern shore had contributions of 1–2 orders of magnitude greater than the southern shore. It identified the Northwestern Basin and the Adriatic Sea as the main exporters to the southern and eastern basins. The ten main contributing rivers accounted for up to 52% of the total inputs, with a decreased contribution of the Nile compared to previous estimates. The re-evaluation of potential contributions between northern and southern basins redistributes roles and responsibilities, emphasizing the need for collective and regional action to limit pollution in river basins before its transfer to marine compartments.

We estimated average residence times for floating MPs at the sea surface to range from 1 to 3 weeks in highly dissipative sub-basins to 11 weeks in the Ionian Sea, and we suggested that the existence of a near steady state of surface concentrations, compensating sources by sinks, would imply a stranding rate much lower than in previous studies, around 60% instead of 90%. At depth, sinking MPs released at the coast were likely to sediment quickly given the nature of the polymers, being mostly trapped on the continental shelves, along the continental slope, or in canyons. This observation was related to the type of sources considered in this study exclusively located on the coastline.

In order to improve flux and stock budget in the Mediterranean Sea, dispersion models need to take better into account the different processes involved in the MP transport from sources to sinks, relying more closely on observations and experimental studies. Future observation campaigns at sea should also use modeled scenarios as a tool for designing the sampling strategies. High-frequency temporal monitoring is particularly needed offshore, along the coast, in river plumes, frontal zones, and eddies. This will help to better understand the temporal variability of measurements associated with weather conditions and sea surface state as well as the temporal variability of the riverine sources.

**Supplementary information** The estimates of MP inputs into the Mediterranean Sea, as considered in this study (549 river basins at 0.08° resolution) and the source file at the global scale from Weiss et al. (2021) (9988 river basins at 0.5°resolution), have been deposited in geospatial vector data for a geographic information system (GIS) and in dataset.csv format on figshare with the identifier doi:10.6084/m9.figshare.26662189 (available at https://figshare.com/articles/dataset/Supplementary_data_for_From_source_to_sink_part_1_characterization_and_Lagrangian_tracking_of_riverine_microplastics_in_the_Mediterranean_Basin_/26662189).

## Data Availability

Not applicable

## Code availability

Code SYMPHONIE available from INSU-certified service SIROCCO (sirocco.obs-mip.fr).

## References

[CR1] Andrady AL (2017) The plastic in microplastics: a review. Mar Pollut Bull 119(1):12–22. 10.1016/j.marpolbul.2017.01.082. Elsevier Ltd 10.1016/j.marpolbul.2017.01.082 28449819

[CR2] Bajon R, Huck T, Grima N et al (2023) Influence of waves on the three-dimensional distribution of plastic in the ocean. Mar Pollut Bull 187. 10.1016/j.marpolbul.2022.114533. Elsevier Ltd 10.1016/j.marpolbul.2022.114533 36610301

[CR3] Baldwin AK, Corsi SR, Mason SA (2016) Plastic debris in 29 great lakes tributaries: relations to watershed attributes and hydrology. Environ Sci Technol 50(19):10377–10385. 10.1021/acs.est.6b02917 27627676 10.1021/acs.est.6b02917

[CR4] Baudena A, Kiko R, Jalón-Rojas I et al (2023) Low-density plastic debris dispersion beneath the Mediterranean Sea surface. Environ Sci Technol 57(19):7503–7515. 10.1021/acs.est.2c08873. American Chemical Society 10.1021/acs.est.2c08873 37125732

[CR5] Baudena A, Ser-Giacomi E, Jalón-Rojas I et al (2022) The streaming of plastic in the Mediterranean Sea. Nat Commun 13(1). 10.1038/s41467-022-30572-5. Springer US 10.1038/s41467-022-30572-5 PMC9142569 35624104

[CR6] Bouffard J, Vignudelli S, Herrmann M et al (2008) Comparison of ocean dynamics with a regional circulation model and improved altimetry in the North-Western Mediterranean. Terrestrial, Atmospheric and Oceanic Sciences 19(1–2):117–133. 10.3319/TAO.2008.19.1-2.117(SA)

[CR7] Bowman D, Manor-Samsonov N, Golik A (1998) Dynamics of litter pollution on Israeli Mediterranean beaches: a budgetary, litter flux approach. J Coastal Res 14(2):418–432

[CR8] Brunner K, Kukulka T, Proskurowski G et al (2015) Passive buoyant tracers in the ocean surface boundary layer: 2. Observations and simulations of microplastic marine debris. J Geophys Res Ocean, pp 1–15. 10.1002/2015JC010840.Received

[CR9] Campbell SH, Williamson PR, Hall BD (2017) Microplastics in the gastrointestinal tracts of fish and the water from an urban prairie creek. Facets 2(1):395–409. 10.1139/facets-2017-0008

[CR10] Chenillat F, Huck T, Maes C et al (2021) Fate of floating plastic debris released along the coasts in a global ocean model. Mar Pollut Bull 165(February):112116. 10.1016/j.marpolbul.2021.112116. Elsevier Ltd 10.1016/j.marpolbul.2021.112116 33581569

[CR11] Chubarenko I, Bagaev A, Zobkov M et al (2016) On some physical and dynamical properties of microplastic particles in marine environment. Mar Pollut Bull 108(1–2):105–112. 10.1016/j.marpolbul.2016.04.048 27184128 10.1016/j.marpolbul.2016.04.048

[CR12] CIESIN (2018) Gridded population of the world, Version 4 (GPWv4): population density, Revision 11 - center for international earth science information network - CIESIN - Columbia University. 10.7927/H49C6VHW. Palisades, NY

[CR13] Constant M, Alary C, Weiss L et al (2023) Trapped microplastics within vertical redeposited sediment: experimental study simulating lake and channeled river systems during resuspension events. Environ Pollut 322(February):121212. 10.1016/j.envpol.2023.121212. Elsevier Ltd 10.1016/j.envpol.2023.121212 36740164

[CR14] Constant M, Ludwig W, Kerhervé P et al (2020) Microplastic fluxes in a large and a small Mediterranean river catchments: the Têt and the Rhône. Northwestern Mediterranean Sea. Sci Total Environ 716. 10.1016/j.scitotenv.2020.136984 10.1016/j.scitotenv.2020.136984 32059309

[CR15] Cózar A, Sanz-Martín M, Martí E et al (2015) Plastic accumulation in the Mediterranean Sea. PLoS ONE 10(4):1–12. 10.1371/journal.pone.0121762 10.1371/journal.pone.0121762 PMC4382178 25831129

[CR16] Damien P, Bosse A, Testor P et al (2017) Modeling postconvective submesoscale coherent vortices in the northwestern Mediterranean Sea. J Geophys Res Ocean. 10.1002/2016JC012114

[CR17] Deocaris CC, Allosada JO, Ardiente LT et al (2019) Occurrence of microplastic fragments in the Pasig River. H2Open J 2(1):92–100. 10.2166/h2oj.2019.001

[CR18] Estournel C (2003) Observation and modeling of the winter coastal oceanic circulation in the Gulf of Lion under wind conditions influenced by the continental orography (FETCH experiment). J Geophys Res 108(C3):8059. 10.1029/2001JC000825

[CR19] Estournel C, Testor P, Damien P et al (2016) High resolution modeling of dense water formation in the north-western Mediterranean during winter 2012–2013: processes and budget. J Geophys Res Ocean 121(7):5367–5392. 10.1002/2016JC011935

[CR20] Estournel C, Mikolajczak G, Ulses C et al (2023) Sediment dynamics in the gulf of lion (NW Mediterranean Sea) during two autumn–winter periods with contrasting meteorological conditions. Prog Oceanogr 210:102942. 10.1016/j.pocean.2022.102942

[CR21] Estournel C, Marsaleix P, Ulses C (2021) A new assessment of the circulation of Atlantic and Intermediate Waters in the Eastern Mediterranean. Prog Oceanogr 198:102673. 10.1016/j.pocean.2021.102673. Elsevier Ltd

[CR22] Fabri-Ruiz S, Baudena A, Moullec F et al (2023) Mistaking plastic for zooplankton: risk assessment of plastic ingestion in the Mediterranean Sea. Sci Total Environ 856. 10.1016/j.scitotenv.2022.159011. Elsevier B.V 10.1016/j.scitotenv.2022.159011 36170920

[CR23] Faure F, Demars C, Wieser O et al (2015) Plastic pollution in Swiss surface waters: nature and concentrations, interaction with pollutants. Environ Chem 12(5):582–591. 10.1071/EN14218

[CR24] Galgani F, Brien ASo, Weis J et al (2021) Are litter, plastic and microplastic quantities increasing in the ocean? Microplastics and Nanoplastics 1(1):8–11. 10.1186/s43591-020-00002-8. Microplastics and Nanoplastics ISBN: 4359102000002

[CR25] GESAMP (2019) Guidelines or the monitoring and assessment of plastic litter and microplastics in the ocean. Rep Stud GESAMP p 130p

[CR26] Geyer R, Jambeck JR, Law KL (2017) Production, use, and fate of all plastics ever made. Sci Adv 3(7):25–29. 10.1126/sciadv.1700782 10.1126/sciadv.1700782 PMC5517107 28776036

[CR27] Guerrini F, Mari L, Casagrandi R (2022) A coupled Lagrangian-Eulerian model for microplastics as vectors of contaminants applied to the Mediterranean Sea. Environ Res Lett 17(2). 10.1088/1748-9326/ac4fd9. IOP Publishing Ltd

[CR28] Guizien K, Belharet M, Marsaleix P et al (2012) Using larval dispersal simulations for marine protected area design: application to the Gulf of Lions (northwest Mediterranean). Limnol Oceanogr 57(4):1099–1112. 10.4319/lo.2012.57.4.1099

[CR29] Haarr ML, Westerveld L, Fabres J et al (2019) A novel GIS-based tool for predicting coastal litter accumulation and optimising coastal cleanup actions. Mar Pollut Bull 139:117–126. 10.1016/j.marpolbul.2018.12.025 30686408 10.1016/j.marpolbul.2018.12.025

[CR30] Harris PT, Maes T, Raubenheimer K et al (2023) A marine plastic cloud - global mass balance assessment of oceanic plastic pollution. Cont Shelf Res 255. 10.1016/j.csr.2023.104947. Elsevier Ltd

[CR31] Hatzonikolakis Y, Giakoumi S, Raitsos DE et al (2022) Quantifying transboundary plastic pollution in marine protected areas across the Mediterranean Sea. Front Mar Sci 8. 10.3389/fmars.2021.762235. Frontiers Media S.A

[CR32] Isachenko I, Khatmullina L, Chubarenko I et al (2016) Settling velocity of marine microplastic particles: laboratory tests. EGUGA, pp EPSC2016–6553

[CR33] Isobe A, Kubo K, Tamura Y et al (2014) Selective transport of microplastics and mesoplastics by drifting in coastal waters. Mar Pollut Bull 89(1–2):324–330. 10.1016/j.marpolbul.2014.09.041 25287228 10.1016/j.marpolbul.2014.09.041

[CR34] Jalón-Rojas I, Wang XH, Fredj E (2019) A 3D numerical model to track marine plastic debris (TrackMPD): sensitivity of microplastic trajectories and fates to particle dynamical properties and physical processes. Mar Pollut Bull 141(February):256–272. 10.1016/j.marpolbul.2019.02.052. Elsevier 10.1016/j.marpolbul.2019.02.052 30955734

[CR35] Jambeck JR, Geyer R, Wilcox C et al (2015) Plastic waste inputs from land into the ocean - supplementary materials. Science 347(6223):768–771. 10.1126/science.1260352, arXiv:1011.1669v3. ISBN: 0036-8075 10.1126/science.1260352 25678662

[CR36] Kaandorp ML, Dijkstra HA, Van Sebille E (2020) Closing the Mediterranean marine floating plastic mass budget: inverse modeling of sources and sinks. Environ Sci Technol 54(19):11980–11989. 10.1021/acs.est.0c01984 32852202 10.1021/acs.est.0c01984 PMC7547878

[CR37] Kaandorp MLA, Lobelle D, Kehl C et al (2023) Global mass of buoyant marine plastics dominated by large long-lived debris. Nat Geosci 16(8):689–694. 10.1038/s41561-023-01216-0

[CR38] Kaiser D, Kowalski N, Waniek JJ (2017) Effects of biofouling on the sinking behavior of microplastics. Environ Res Lett 12(12). 10.1088/1748-9326/aa8e8b

[CR39] Kane IA, Clare MA (2019) Dispersion, accumulation and the ultimate fate of microplastics in deep-marine environments: a review and future directions. Front Earth Sci 7(April):80. 10.3389/FEART.2019.00080

[CR40] Kataoka T, Nihei Y, Kudou K et al (2019) Assessment of the sources and inflow processes of microplastics in the river environments of Japan. Environ Pollut 244:958–965. 10.1016/j.envpol.2018.10.111 30469290 10.1016/j.envpol.2018.10.111

[CR41] Kedzierski M, Palazot M, Soccalingame L et al (2022) Chemical composition of microplastics floating on the surface of the Mediterranean Sea. Marine Pollution Bulletin 174(January). 10.1016/j.marpolbul.2021.113284 10.1016/j.marpolbul.2021.113284 34995887

[CR42] Khatmullina L, Isachenko I (2017) Settling velocity of microplastic particles of regular shapes. Mar Pollut Bull 114(2):871–880. 10.1016/j.marpolbul.2016.11.024 27863879 10.1016/j.marpolbul.2016.11.024

[CR43] Kooi M, Reisser J, Slat B et al (2016) The effect of particle properties on the depth profile of buoyant plastics in the ocean. Sci Rep 6(October):1–10. 10.1038/srep33882 27721460 10.1038/srep33882 PMC5056413

[CR44] Kukulka T, Proskurowski G, Morét-Ferguson S et al (2012) The effect of wind mixing on the vertical distribution of buoyant plastic debris. Geophys Res Lett 39(7):1–6. 10.1029/2012GL051116

[CR45] Kukulka T, Brunner K (2015) Passive buoyant tracers in the ocean surface boundary layer: 1. Influence of equilibrium wind-waves on vertical distributions. J Geophysical Research: Oceans 120(5):3837–3858. Wiley Online Library

[CR46] Lahens L, Strady E, Kieu-Le TC et al (2018) Macroplastic and microplastic contamination assessment of a tropical river (Saigon River, Vietnam) transversed by a developing megacity. Environ Pollut 236:661–671. 10.1016/J.ENVPOL.2018.02.005. Elsevier 10.1016/j.envpol.2018.02.005 29438952

[CR47] Lebreton LC, Van Der Zwet J, Damsteeg JW et al (2017) River plastic emissions to the world’s oceans. Nat Commun 8:1–10. 10.1038/ncomms15611. Nature Publishing Group 10.1038/ncomms15611 PMC5467230 28589961

[CR48] Liubartseva S, Coppini G, Lecci R et al (2018) Tracking plastics in the Mediterranean: 2D Lagrangian model. Mar Pollut Bull 129(1):151–162. 10.1016/J.MARPOLBUL.2018.02.019. Pergamon 10.1016/j.marpolbul.2018.02.019 29680533

[CR49] Ludwig W, Dumont E, Meybeck M et al (2009) River discharges of water and nutrients to the Mediterranean and Black Sea: major drivers for ecosystem changes during past and future decades? Prog Oceanogr 80(3–4):199–217. 10.1016/j.pocean.2009.02.001

[CR50] Macias D, Cózar A, Garcia-Gorriz E et al (2019) Surface water circulation develops seasonally changing patterns of floating litter accumulation in the Mediterranean Sea. A modelling approach. Marine Pollution Bulletin 149(September):110619. 10.1016/j.marpolbul.2019.110619. Elsevier

[CR51] Macias D, Stips A, Hanke G (2022) Model based estimate of transboundary litter pollution on Mediterranean coasts. Mar Pollut Bull 175. 10.1016/j.marpolbul.2021.113121. Elsevier Ltd 10.1016/j.marpolbul.2021.113121 34839956

[CR52] Mani T, Hauk A, Walter U et al (2015) Microplastics profile along the Rhine River. Sci Rep 5(December):1–7. 10.1038/srep17988 10.1038/srep17988 PMC4672315 26644346

[CR53] Mansui J, Molcard A, Ourmières Y (2015) Modelling the transport and accumulation of floating marine debris in the Mediterranean basin. Mar Pollut Bull 91(1):249–257. 10.1016/j.marpolbul.2014.11.037 25534631 10.1016/j.marpolbul.2014.11.037

[CR54] Mansui J, Darmon G, Ballerini T et al (2020) Predicting marine litter accumulation patterns in the Mediterranean basin : spatio-temporal variability and comparison with empirical data. Progress in Oceanography, p 102268. 10.1016/j.pocean.2020.102268. Elsevier Ltd

[CR55] Many G, Ulses C, Estournel C et al (2021) Particulate organic carbon dynamics in the Gulf of Lion shelf (NW Mediterranean) using a coupled hydrodynamic-biogeochemical model. Biogeosciences 18(19):5513–5538. 10.5194/bg-18-5513-2021

[CR56] Marsaleix P, Auclair F, Estournel C (2006) Considerations on open boundary conditions for regional and coastal ocean models. J Atmos Oceanic Tech 23(11):1604–1613. 10.1175/JTECH1930.1

[CR57] Marsaleix P, Auclair F, Floor JW et al (2008) Energy conservation issues in sigma-coordinate free-surface ocean models. Ocean Model 20(1):61–89. 10.1016/j.ocemod.2007.07.005

[CR58] McWilliams JC, Restrepo JM (1999) The wave-driven ocean circulation. J Phys Oceanogr 29(10):2523–2540. 10.1175/1520-0485(1999)029<2523:TWDOC>2.0.CO;2. American Meteorological Society Place: Boston MA, USA

[CR59] Michaud H, Marsaleix P, Leredde Y et al (2012) Three-dimensional modelling of wave-induced current from the surf zone to the inner shelf. Ocean Sci 8(4):657–681. 10.5194/os-8-657-2012

[CR60] Mikolajczak G, Estournel C, Ulses C et al (2020) Impact of storms on residence times and export of coastal waters during a mild autumn/winter period in the Gulf of Lion. Cont Shelf Res 207(May). 10.1016/j.csr.2020.104192

[CR61] Nguyen-Duy T, Ayoub NK, Marsaleix P et al (2021) Variability of the red river plume in the gulf of Tonkin as revealed by numerical modeling and clustering analysis. Front Mar Sci 8(November):1–25. 10.3389/fmars.2021.77213935685121

[CR62] Onink V, Wichmann D, Delandmeter P et al (2019) The role of Ekman currents, geostrophy, and stokes drift in the accumulation of floating microplastic. J Geophys Res: Oceans 124(3):1474–1490. 10.1029/2018JC014547 31218155 10.1029/2018JC014547 PMC6559306

[CR63] Onink V, Kaandorp MLA, van Sebille E et al (2022) Influence of particle size and fragmentation on large-scale microplastic transport in the mediterranean sea. Environ Sci Technol 56(22):15528–15540. 10.1021/acs.est.2c03363 36270631 10.1021/acs.est.2c03363 PMC9671120

[CR64] Pedrotti ML, Petit S, Elineau A et al (2016) Changes in the floating plastic pollution of the mediterranean sea in relation to the distance to land. PLoS ONE 11(8):1–14. 10.1371/journal.pone.0161581 10.1371/journal.pone.0161581 PMC4996504 27556233

[CR65] Pedrotti ML, Lombard F, Baudena A et al (2022) An integrative assessment of the plastic debris load in the Mediterranean Sea. Sci Total Environ 838:155958. 10.1016/j.scitotenv.2022.155958 35580673 10.1016/j.scitotenv.2022.155958

[CR66] Poulain M, Mercier MJ, Brach L et al (2019) Small microplastics as a main contributor to plastic mass balance in the North Atlantic subtropical gyre. Environ Sci Technol 53(3):1157–1164. 10.1021/acs.est.8b05458 30575384 10.1021/acs.est.8b05458

[CR67] Rétif F (2015) Modélisation du niveau instantané de la mer en conditions paroxysmales: Caractérisation des contributions à différentes échelles de temps et d’espace. PhD thesis, Université de Montpellier

[CR68] Sadaoui M, Ludwig W, Bourrin F et al (2018) The impact of reservoir construction on riverine sediment and carbon fluxes to the Mediterranean Sea. Prog Oceanogr 163(July):94–111. 10.1016/j.pocean.2017.08.003

[CR69] Sadri SS, Thompson RC (2014) On the quantity and composition of floating plastic debris entering and leaving the Tamar Estuary, Southwest England. Mar Pollut Bull 81(1):55–60. 10.1016/j.marpolbul.2014.02.020 24613232 10.1016/j.marpolbul.2014.02.020

[CR70] Schroeder K, Garcìa-Lafuente J, Josey SA et al (2012) Circulation of the mediterranean sea and its variability. In: Lionello P (ed) The climate of the Mediterranean region. Elsevier, pp 187–256

[CR71] Simon-Sánchez L, Grelaud M, Garcia-Orellana J et al (2019) River Deltas as hotspots of microplastic accumulation: the case study of the Ebro River (NW Mediterranean). Sci Total Environ 687:1186–1196. 10.1016/j.scitotenv.2019.06.168 31412454 10.1016/j.scitotenv.2019.06.168

[CR72] Sonke JE, Koenig AM, Yakovenko N et al (2022) A mass budget and box model of global plastics cycling, degradation and dispersal in the land-ocean-atmosphere system. Microplastics and Nanoplastics 2(1). 10.1186/s43591-022-00048-w. Springer Science and Business Media LLC

[CR73] Soto-Navarro J, Jordá G, Deudero S et al (2020) 3D hotspots of marine litter in the Mediterranean: a modeling study. Mar Pollut Bull 155(April):111159. 10.1016/j.marpolbul.2020.111159. Elsevier 10.1016/j.marpolbul.2020.111159 32469776

[CR74] Stokes GG (1851) On the effect of the internal friction of fluids on the motion of pendulums, vol 9. Pitt Press Cambridge

[CR75] Suaria G, Avio CG, Mineo A et al (2016) The Mediterranean plastic soup: synthetic polymers in Mediterranean surface waters. Sci Rep 6:1–10. 10.1038/srep37551 27876837 10.1038/srep37551 PMC5120331

[CR76] Tan X, Yu X, Cai L et al (2019) Microplastics and associated PAHs in surface water from the Feilaixia Reservoir in the Beijiang River, China. Chemosphere, pp 834–840. 10.1016/j.chemosphere.2019.01.022 10.1016/j.chemosphere.2019.01.022 30684781

[CR77] Tolman HL (2009) User manual and system documentation of WAVEWATCH III TM version 3.14. Technical note, MMAB Contribution 276:220

[CR78] Tsiaras K, Costa E, Morgana S et al (2022) Microplastics in the Mediterranean: variability from observations and model analysis. Front Mar Sci 9. 10.3389/fmars.2022.784937. Frontiers Media S.A

[CR79] Ulses C, Estournel C, Fourrier M et al (2021) Oxygen budget of the north-western Mediterranean deep- convection region. Biogeosciences 18(3):937–960. 10.5194/bg-18-937-2021

[CR80] van der Wal M, van der Meulen M, Tweehuijsen G et al (2015) Identification and assessment of riverine input of (marine) litter. Tech. Rep, April, European Commission

[CR81] van Sebille E, Aliani S, Law KL et al (2020) The physical oceanography of the transport of floating marine debris. Environ Res Lett 15(2). 10.1088/1748-9326/ab6d7d

[CR82] van Sebille E, Wilcox C, Lebreton LC et al (2015) A global inventory of small floating plastic debris. Environ Res Lett 10(12):124006. 10.1088/1748-9326/10/12/124006. IOP Publishing

[CR83] Vousdoukas MI, Ranasinghe R, Mentaschi L et al (2020) Sandy coastlines under threat of erosion. Nat Clim Chang 10(3):260–263. 10.1038/s41558-020-0697-0

[CR84] Waldschläger K, Schüttrumpf H (2019) Effects of particle properties on the settling and rise velocities of microplastics in freshwater under laboratory conditions. Environ Sci Technol 53(4):1958–1966. 10.1021/acs.est.8b06794 30688437 10.1021/acs.est.8b06794

[CR85] Weiss L, Ludwig W, Heussner S et al (2021) The missing ocean plastic sink: gone with the rivers. Science 373(6550):107–111. 10.1126/science.abe0290 34210886 10.1126/science.abe0290

[CR86] Xiong X, Wu C, Elser JJ et al (2019) Occurrence and fate of microplastic debris in middle and lower reaches of the Yangtze River – From inland to the sea. Sci Total Environ 659:66–73. 10.1016/j.scitotenv.2018.12.313 30597469 10.1016/j.scitotenv.2018.12.313

[CR87] Zambianchi E, Trani M, Falco P (2017) Lagrangian transport of marine litter in the Mediterranean sea. Front Environ Sci 5. 10.3389/fenvs.2017.00005. Frontiers Media SA

[CR88] Zhang H (2017) Transport of microplastics in coastal seas. Estuar Coast Shelf Sci 199:74–86. 10.1016/j.ecss.2017.09.032

[CR89] Zhiyao S, Tingting W, Fumin X et al (2008) A simple formula for predicting settling velocity of sediment particles. Water Science and Engineering 1(1):37–43. 10.1016/s1674-2370(15)30017-x. Hohai University. Production and hosting by Elsevier B.V

